# Deciphering the STAT3-PXN positive feedback loop in GBM, IDH-wildtype: transcriptional regulation and inhibition of YB-1 ubiquitination

**DOI:** 10.1038/s41420-026-03035-9

**Published:** 2026-03-23

**Authors:** Xiaodong Li, Hongyan Guo, Ziyi Liu, Tianze Wang, Maode Wang, Wei Chen, Hai Yu

**Affiliations:** 1 Department of Neurosurgery, The First Affiliated Hospital of Xi’an Jiaotong University, Xi’an, China; 2 Center of Brain Science, The First Affiliated Hospital of Xi’an Jiaotong University, Xi’an, China; 3 Key Laboratory of Xinjiang Endemic and Ethnic Diseases, Ministry of Education, Shihezi University School of Medicine, Shihezi, China; 4 Department of Pathophysiology, Shihezi University School of Medicine, Shihezi, China

**Keywords:** CNS cancer, Mechanisms of disease

## Abstract

Glioblastoma (GBM) is the most fatal primary brain malignancy in adults, with a median survival of approximately 15 months. The 2021 WHO classification redefined GBM as exclusively IDH-wildtype based on its characteristic molecular and clinical features. In this study, we aimed to identify key prognostic genes in GBM, IDH-wildtype. Using univariate Cox proportional hazards regression analysis, PXN was identified as a critical upregulated gene in GBM, IDH-wildtype, significantly associated with poor prognosis. Its expression was further validated by qRT-PCR, western blotting, and immunohistochemistry. Functional assays revealed that elevated PXN enhances GBM malignancy, whereas its knockdown suppresses corresponding malignant features. Mechanistically, PXN and STAT3 form a positive feedback loop: STAT3 upregulates PXN transcription, and PXN, in turn, activates STAT3 by regulating SRC transcription. Additionally, PXN stabilizes YB-1 protein by inhibiting its ubiquitination. Further mRNA sequencing analysis demonstrated that YB-1 contributes to maintaining GBM malignancy through multiple signaling pathways. These results suggest that the STAT3-PXN positive feedback axis and the regulation of YB-1 stability by PXN may offer novel targets for GBM therapy.

**Figure:**
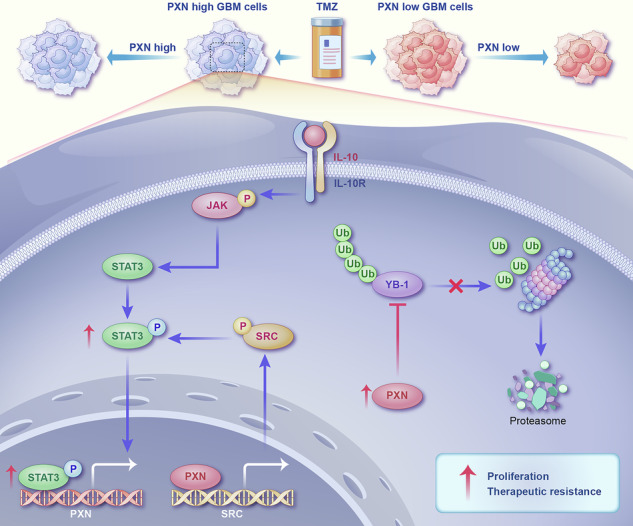
PXN is elevated in GBM, IDH-wildtype and associated with poor prognosis and malignant features. STAT3 directly promotes PXN transcription, and PXN reciprocally activates STAT3 by regulating SRC transcription. PXN stabilizes YB-1 protein by inhibiting its ubiquitin-mediated degradation.

PXN is elevated in GBM, IDH-wildtype and associated with poor prognosis and malignant features. STAT3 directly promotes PXN transcription, and PXN reciprocally activates STAT3 by regulating SRC transcription. PXN stabilizes YB-1 protein by inhibiting its ubiquitin-mediated degradation.

## Introduction

Glioblastoma (GBM) is the most common and fatal primary brain tumor in adults [1]. In 2021, the fifth edition of the WHO Classification of Tumors of the Central Nervous System (WHO CNS5) redefined GBM as exclusively isocitrate dehydrogenase (IDH)-wildtype, distinguishing it from IDH-mutant astrocytomas based on its unique molecular and clinical features [2]. Current multimodal approaches for GBM include maximal surgical resection, followed by concurrent and adjuvant chemo-radiotherapy [3]. Despite these efforts, the prognosis for GBM patients remains disheartening. Studies have reported that patients with GBM, IDH-wildtype have a median overall survival of 12-21 months [4]. Therapeutic resistance, particularly to temozolomide (TMZ), contributes to this grim scenario [5]. TMZ has served as the first-line chemotherapeutic agent for newly diagnosed glioblastoma for over two decades [6], yet nearly all patients eventually develop resistance to TMZ [7]. Therefore, focusing on GBM, IDH-wildtype is essential to elucidate the mechanisms underlying tumor progression and therapeutic response.

Focal adhesions (FAs) are large, integrin-containing, multi-protein complexes that span the plasma membrane, acting as a liaison between the cellular cytoskeleton and extracellular matrix (ECM) [8]. Paxillin (PXN), a multifunctional adaptor protein, recruits structural and signaling molecules to FAs, where it transduces adhesion and growth factor signals to regulate cell shape, adhesion, motility, and gene expression [9–11]. PXN is frequently overexpressed in multiple cancers, including prostate cancer [12], colorectal cancer [13], and non-small cell lung cancer [14]. Its upregulation is associated with increased malignancy and poor patient prognosis [13, 15, 16]. It has also been implicated in conferring cisplatin resistance in non-small cell lung cancer [17]. In glioma, numerous studies have focused on PXN’s role in focal adhesions. These studies indicate that PXN is often regulated indirectly through other pathways, affecting cytoskeletal reorganization, migration, and invasion [18–23]. Systematic investigations of PXN itself in glioma, however, are largely limited to bioinformatic predictions and basic functional validations [24, 25]. The upstream transcriptional regulation, nuclear roles, and post-translational functions of PXN in GBM have yet to be fully elucidated.

In our study, we first identified PXN as a prognostic factor in GBM, IDH-wildtype through integrative bioinformatic analyses. Subsequent in vitro and in vivo experiments showed that PXN drives malignant phenotypes of GBM cells. We next explored the upstream regulation of PXN, and identified signal transducer and activator of transcription 3 (STAT3) as a key transcriptional regulator. Mechanistic analyses uncovered a STAT3-PXN-SRC-STAT3 positive feedback loop, in which STAT3 transcriptionally upregulates PXN, and nuclear PXN further promotes proto-oncogene tyrosine-protein kinase Src (SRC) transcription, thereby activating STAT3 signaling. Finally, we explored PXN-associated downstream factors and found that PXN stabilizes Y-box binding protein 1 (YB-1) by inhibiting its ubiquitin-mediated degradation. In general, these findings define a STAT3-PXN-SRC-STAT3 positive feedback loop and a PXN-YB-1 regulatory axis in GBM, schematically summarized in the Graphic Abstract.

## Results

### PXN expression is highly elevated in GBM, IDH-wildtype

**Fig. 1:**
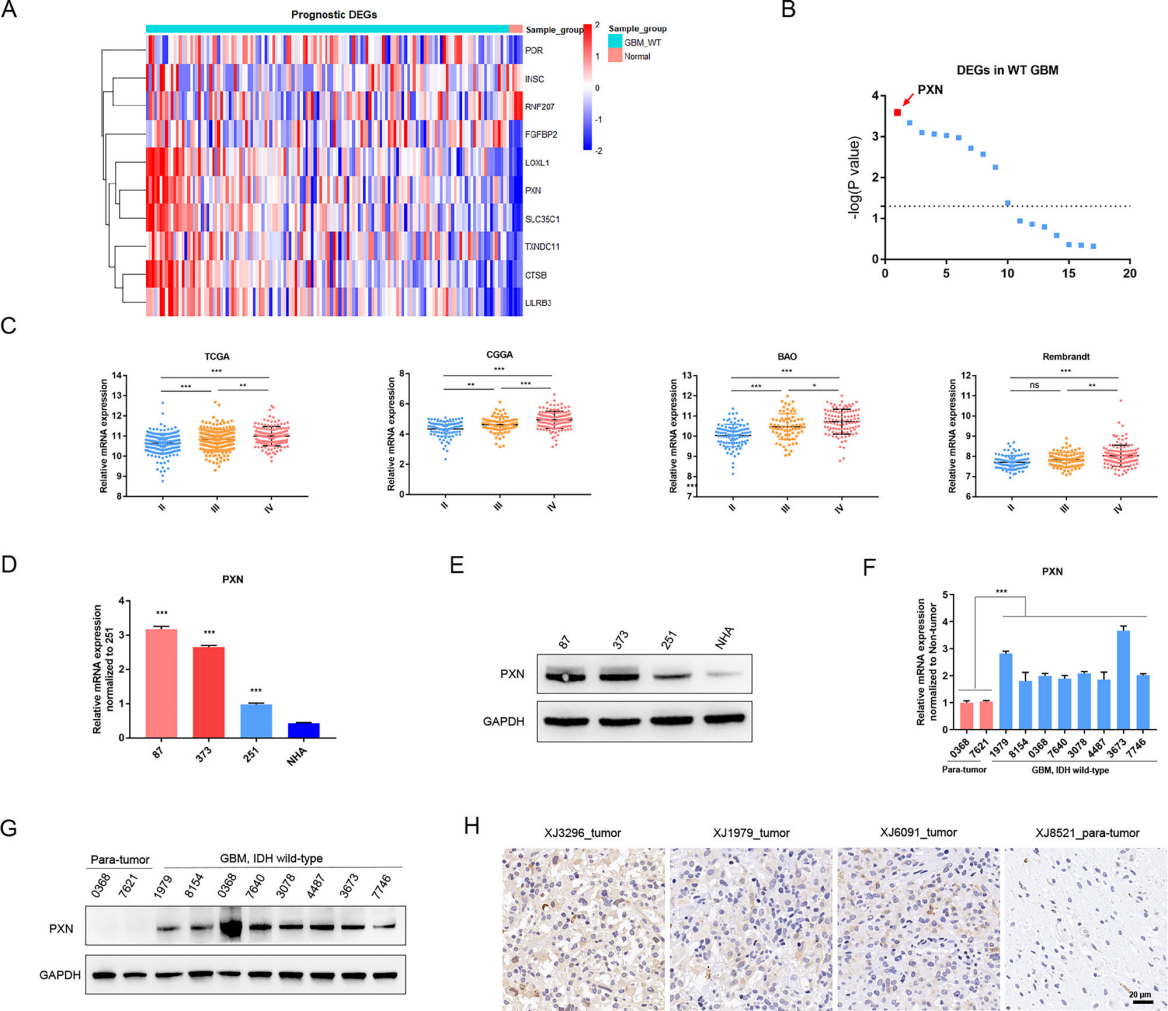
PXN expression is highly elevated in GBM, IDH-wildtype. **A** A heatmap displaying DEGs between GBM, IDH-wildtype and normal samples in TCGA dataset. **B** Comparative analysis showing DEGs most closely related with the prognosis of GBM, IDH-wildtype patients. **C** PXN mRNA expression in grade II, III and IV gliomas using the information from TCGA, CGGA, Rembrandt and BAO datasets. Data are presented as mean ± SD. Statistical significance was determined by one-way ANOVA with Tukey’s post hoc test (ns, not significant; *P < 0.05, **P < 0.01, ***P < 0.001). **D** qRT-PCR detecting PXN mRNA expression in U87, U373 and U251 GBM cells and NHA cells. GAPDH served as a control. Data are presented as mean ± SD of three independent biological replicates. Statistical significance was determined by one-way ANOVA with Dunnett’s multiple comparisons test (***P < 0.001). **E** Western blotting determining PXN protein expression in U87, U373, U251, and NHA cells. **F** qRT-PCR detecting PXN mRNA expression in GBM, IDH-wildtype and para-tumor tissues. GAPDH served as a control. Data are presented as mean ± SD of three independent biological replicates. Statistical significance was determined by a two-tailed Student’s *t*-test (***P < 0.001). **G** Western blotting determining PXN protein expression in GBM, IDH-wildtype and para-tumor tissues. **H** IHC detecting PXN protein expression in GBM, IDH-wildtype and para-tumor tissues. Scale bar = 20 μm.

The dismal clinical prognosis of GBM, IDH-wildtype prompted us to examine the prognosis-related genes in this subtype via univariate Cox proportional hazards regression analysis. As a result, 17 genes were identified in total, with 12 genes showing high expression in tumor tissues. The 10 differentially expressed genes (DEGs) between GBM, IDH-wildtype and normal samples were shown (Fig. 1A). Comparative analysis showed that PXN is one of the most remarkably elevated prognosis-related genes in GBM, IDH-wildtype (Fig. 1B). To verify PXN expression patterns in gliomas, we compared PXN mRNA expression across glioma grades using data from four datasets, including TCGA, CGGA, Rembrandt, and BAO. The results indicated that WHO grade IV glioma exhibits the highest PXN mRNA expression (Fig. 1C). To validate these results in vitro, qRT-PCR and western blotting were performed. The results demonstrated that PXN mRNA and protein expression levels are significantly upregulated in GBM cells compared with normal human astrocytes (NHA), especially in U87 and U373 cells (Fig. 1D, E). Consistently, PXN expression is highly elevated in GBM, IDH-wildtype tissues compared with para-tumor tissues (Fig. 1F–H). Overall, these findings indicate that PXN is preferentially expressed in GBM, IDH-wildtype, and correlates with poor prognosis of patients.

### Silencing of PXN hinders malignant features of GBM cells

**Fig. 2:**
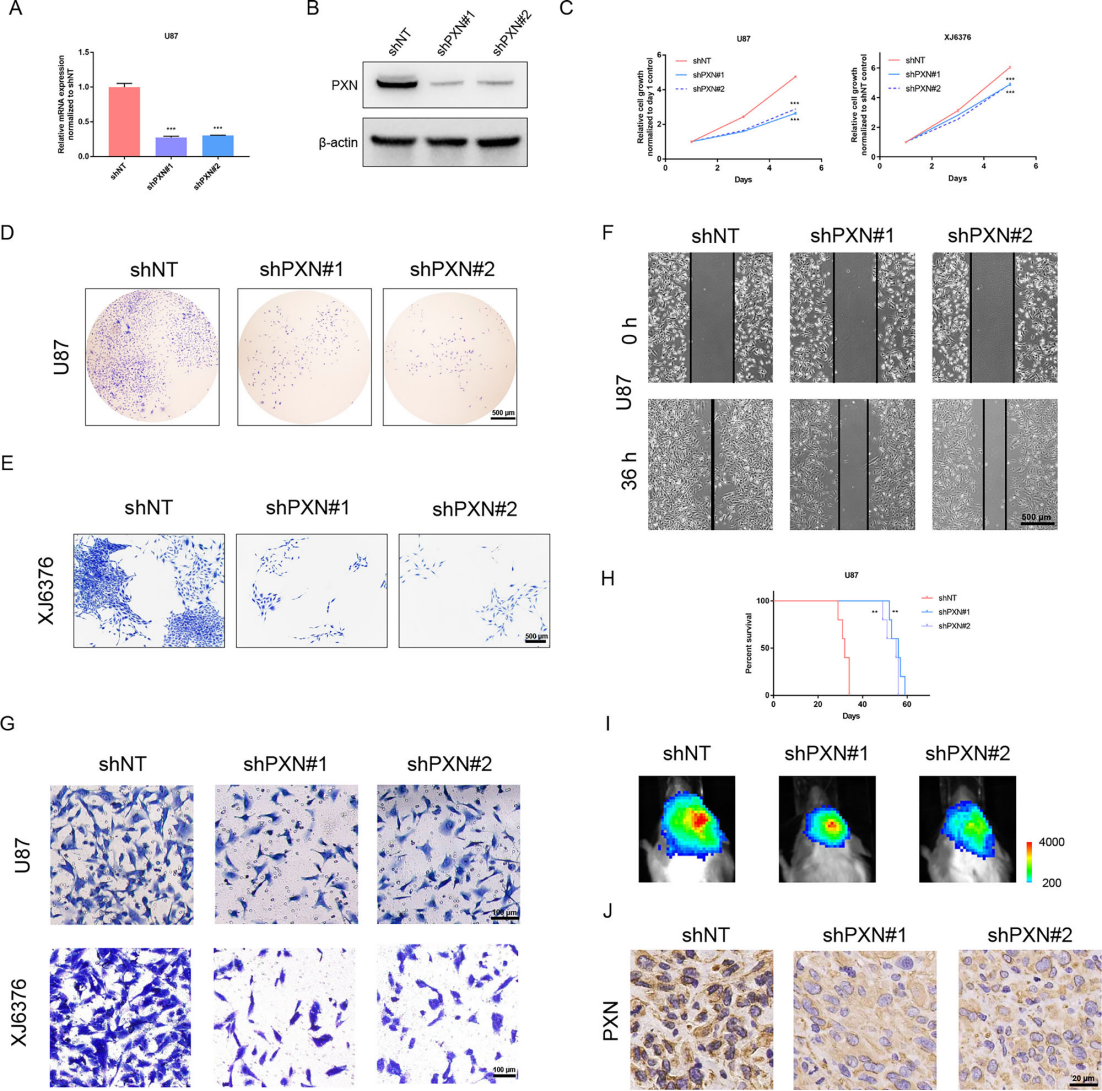
PXN silencing hinders the proliferation, migration and tumorigenicity of GBM cells. **A** qRT-PCR detecting PXN mRNA expression in U87 cells transfected with NT-shRNA or PXN-shRNA. GAPDH served as a control. Data are presented as mean ± SD of three independent biological replicates. Statistical significance was determined by one-way ANOVA with Dunnett’s multiple comparisons test (***P < 0.001). **B** Western blotting determining PXN protein expression in U87 cells transfected with NT-shRNA or PXN-shRNA. **C** Cell viability assay detecting the proliferation ability of U87 and XJ6376 cells transfected with NT-shRNA or PXN-shRNA. Data are presented as mean ± SD of three independent biological replicates. Statistical significance was determined by one-way ANOVA with Dunnett’s multiple comparisons test (***P < 0.001). **D**, **E** Colony formation assay detecting tumorigenic potential of U87 (**D**) and XJ6376 (**E**) cells transfected with NT-shRNA or PXN-shRNA. Scale bar = 500 μm. **F** Wound healing assay detecting the migratory ability of U87 cells transfected with NT-shRNA or PXN-shRNA. Scale bar = 500 μm. **G** Transwell assay detecting migratory ability of U87 and XJ6376 cells transfected with NT-shRNA or PXN-shRNA. Scale bar = 100 μm. **H** K-M analysis comparing overall survival of mice intracranially injected with U87 cells transfected with either NT-shRNA or PXN-shRNA. Data are presented as median survival time (*n* = 5 mice per group). Statistical significance was determined by log-rank test (**P < 0.01). **I** Representative BLIs of mice injected with luciferase-labeled U87 cells transfected with NT-shRNA or PXN-shRNA. **J** Representative IHC images of mice brain tissues detecting PXN expression in shNT and shPXN groups. Scale bar = 20 μm.

To investigate PXN’s role in GBM, shRNAs targeting PXN (shPXN#1 and shPXN#2) were introduced into U87, U373, and XJ6376 cells. The efficacy of silencing was confirmed via qRT-PCR and western blotting (Fig. 2A, B and S1A, B). Next, we investigated the impact of PXN silencing on GBM cell functions. Cell viability assays revealed that silencing of PXN significantly reduces cell growth (Fig. 2C and S1C). Colony formation assays showed that PXN silencing dramatically abolishes self-renewal ability of GBM cells (Fig. 2D, E and S1D). Transwell and wound healing assays indicated that the migration ability of GBM cells is remarkably impeded by PXN silencing (Fig. 2F, G and S1E, F). To investigate PXN silencing’s in vivo role, we constructed intracranial xenograft tumor models. K-M analysis showed that PXN silencing significantly prolongs the survival of mice (Fig. 2H). Consistently, Bioluminescent imaging (BLI) showed that PXN silencing attenuates the tumor formation ability (Fig. 2I), further evidenced by decreased PXN expression in the shPXN groups using IHC staining of mouse brain tissues (Fig. 2J).

### Overexpression of PXN enhances malignant progression of GBM cells

**Fig. 3:**
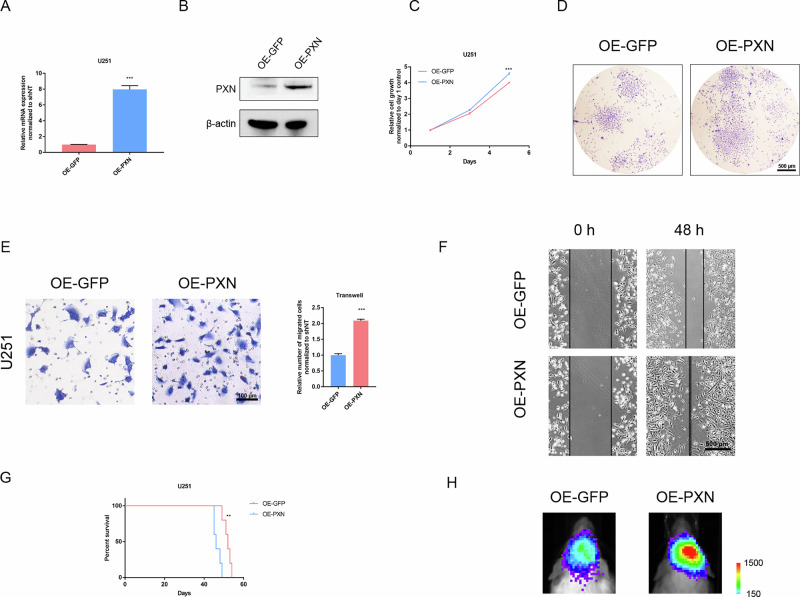
Overexpression of PXN enhances multiple malignant behaviors of GBM cells. **A** qRT-PCR detecting PXN mRNA expression in U251 GBM cells transfected with OE-GFP or OE-PXN lentiviruses. GAPDH served as a control. Data are presented as mean ± SD of three independent biological replicates. Statistical significance was determined by a two-tailed Student’s *t*-test (***P < 0.001). **B** Western blotting determining PXN protein expression in U251 GBM cells transfected with OE-GFP or OE-PXN lentiviruses. **C** Cell viability assay detecting proliferation ability of U251 GBM cells transfected with either OE-GFP or OE-PXN lentiviruses. Data are presented as mean ± SD of three independent biological replicates. Statistical significance was determined by a two-tailed Student’s *t*-test (***P < 0.001). **D** Colony formation assay detecting tumorigenic potential of U251 GBM cells transfected with OE-GFP or OE-PXN lentiviruses. Scale bar = 500 μm. **E** Transwell assay detecting migratory ability of U251 GBM cells transfected with OE-GFP or OE-PXN lentiviruses. Data are presented as mean ± SD of three independent biological replicates. Statistical significance was determined by a two-tailed Student’s *t*-test (***P < 0.001). Scale bar = 100 μm. **F** Wound healing assay detecting migratory ability of U251 GBM cells transfected with OE-GFP or OE-PXN lentiviruses. Scale bar = 500 μm. **G** K-M analysis comparing overall survival of mice intracranially injected with U251 GBM cells transfected with OE-GFP or OE-PXN lentiviruses. Data are presented as median survival time (*n* = 5 mice per group). Statistical significance was determined by log-rank test (**P < 0.01). **H** Representative BLIs of mice intracranially injected with luciferase-labeled U251 GBM cells transfected with OE-GFP or OE-PXN lentiviruses.

To further verify PXN’s oncogenic role in GBM, we subjected GBM cells to overexpression of PXN (OE-PXN) lentiviruses. Compared with U87 and U373 cells, the PXN expression in U251 GBM cells is relatively low (Fig. 1D, E). Thus, PXN overexpressed U251 cells were constructed via lentivirus transfection. qRT-PCR and western blotting confirmed effective upregulation of PXN (Fig. 3A, B). Cell viability assays showed that elevated PXN significantly promotes the proliferation of U251 cells (Fig. 3C). Colony formation assays revealed that PXN overexpression promotes self-renewal ability (Fig. 3D). Transwell and wound healing assays indicated that PXN overexpression remarkably boosts the migration ability of U251 cells (Fig. 3E, F). Additionally, mice injected with OE-PXN U251 cells exhibit worse outcomes than those injected with GFP-overexpressing (OE-GFP) U251 cells (Fig. 3G). BLI showed that PXN overexpression enhances tumor formation ability in vivo (Fig. 3H). These results underscore the functional requirement of PXN in driving multiple malignant behaviors in GBM cells.

### PXN promotes TMZ resistance of GBM cells

**Fig. 4:**
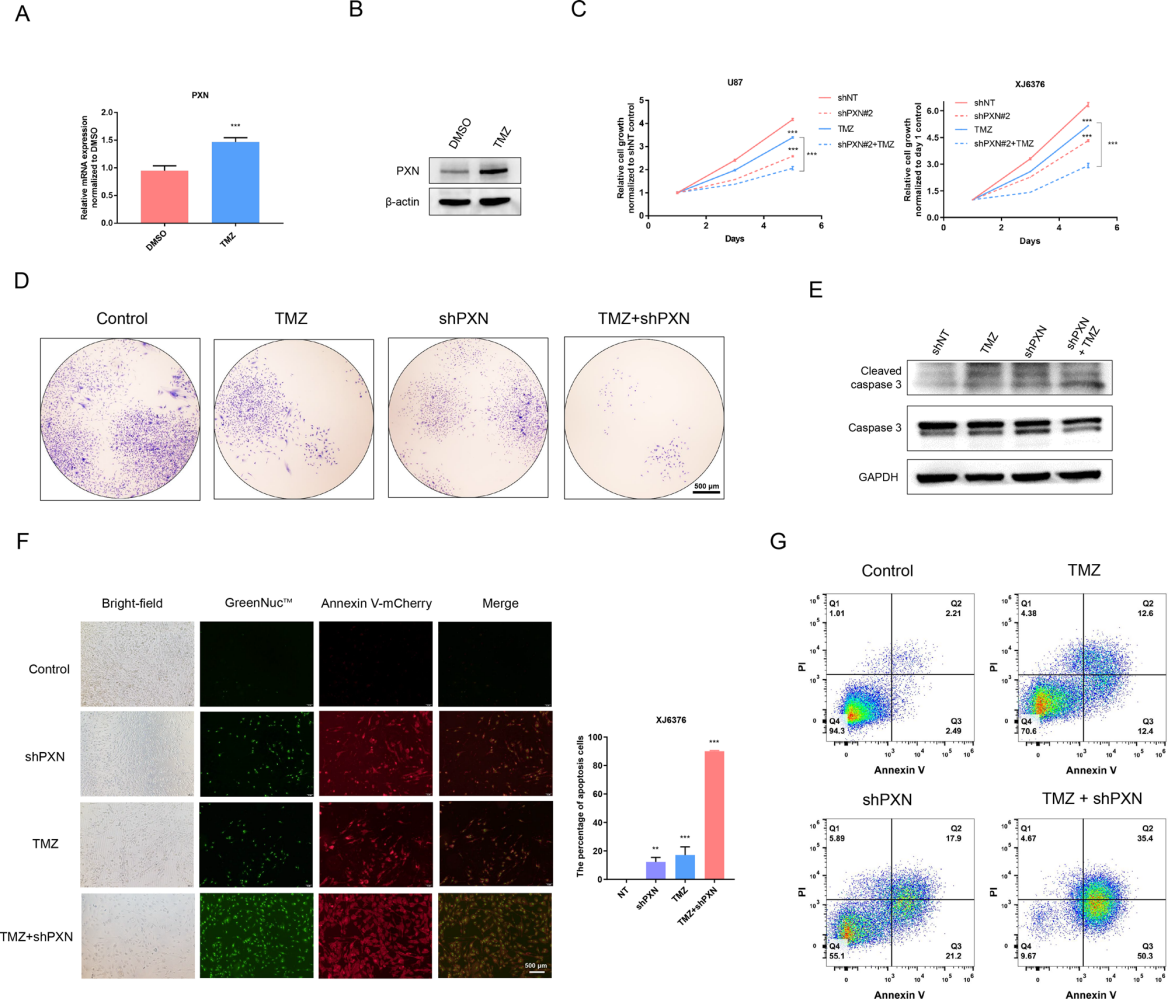
Combined PXN silencing and TMZ treatment enhances apoptosis in GBM cells. **A** qRT-PCR detecting PXN mRNA expression in U87 cells pre-treated with DMSO or TMZ (MCE, HY-17364). Data are presented as mean ± SD of three independent biological replicates. Statistical significance was determined by a two-tailed Student’s *t*-test (***P < 0.001). **B** Western blotting determining PXN protein expression in U87 cells pre-treated with DMSO or TMZ. **C** Cell viability assay detecting proliferation ability of U87 and XJ6376 cells subjected to the indicated treatments. Data are presented as mean ± SD of three independent biological replicates. Statistical significance was determined by a one-way ANOVA with Tukey’s post hoc test (***P < 0.001). **D** Colony formation assay detecting tumorigenic potential of U87 cells subjected to the indicated treatments. Scale bar = 500 μm. **E** Western blotting determining cleaved caspase-3 protein expression in U87 cells subjected to the indicated treatments. **F** In situ fluorescence showing caspase-3 activity in living XJ6376 cells. Data are presented as mean ± SD of three independent biological replicates. Statistical significance was determined by a one-way ANOVA with Dunnett’s multiple comparisons test (**P < 0.01, ***P < 0.001). **G** Flow cytometry analysis for apoptotic rates in U87 cells subjected to the indicated treatments.

To explore the clinical application of targeting PXN, we investigated its response to TMZ treatment. Through qRT-PCR and western blotting, we observed a significant increase in PXN mRNA and protein levels after TMZ exposure (Fig. 4A, B and S2A, B). Cell viability assays showed that PXN knockdown alongside TMZ reduces cell proliferation (Fig. 4C and S2C). Colony formation assays showed impaired self-renewal ability in GBM cells with combined PXN knockdown and TMZ treatment (Fig. 4D). In addition, PXN silencing combined with TMZ increases the expression of cleaved caspase-3 (Fig. 4E, F), indicating a potential for enhanced apoptosis of GBM cells. Further investigation via flow cytometry demonstrated a significant increase in early (AV + ; PI-) and late (AV + ; PI + ) apoptosis in GBM cells with combination therapy (Fig. 4G). These findings highlight the potential of targeting PXN in combination with TMZ as a therapeutic modality for GBM.

### STAT3 acts as an upstream regulator of PXN to drive the malignancy of GBM

**Fig. 5:**
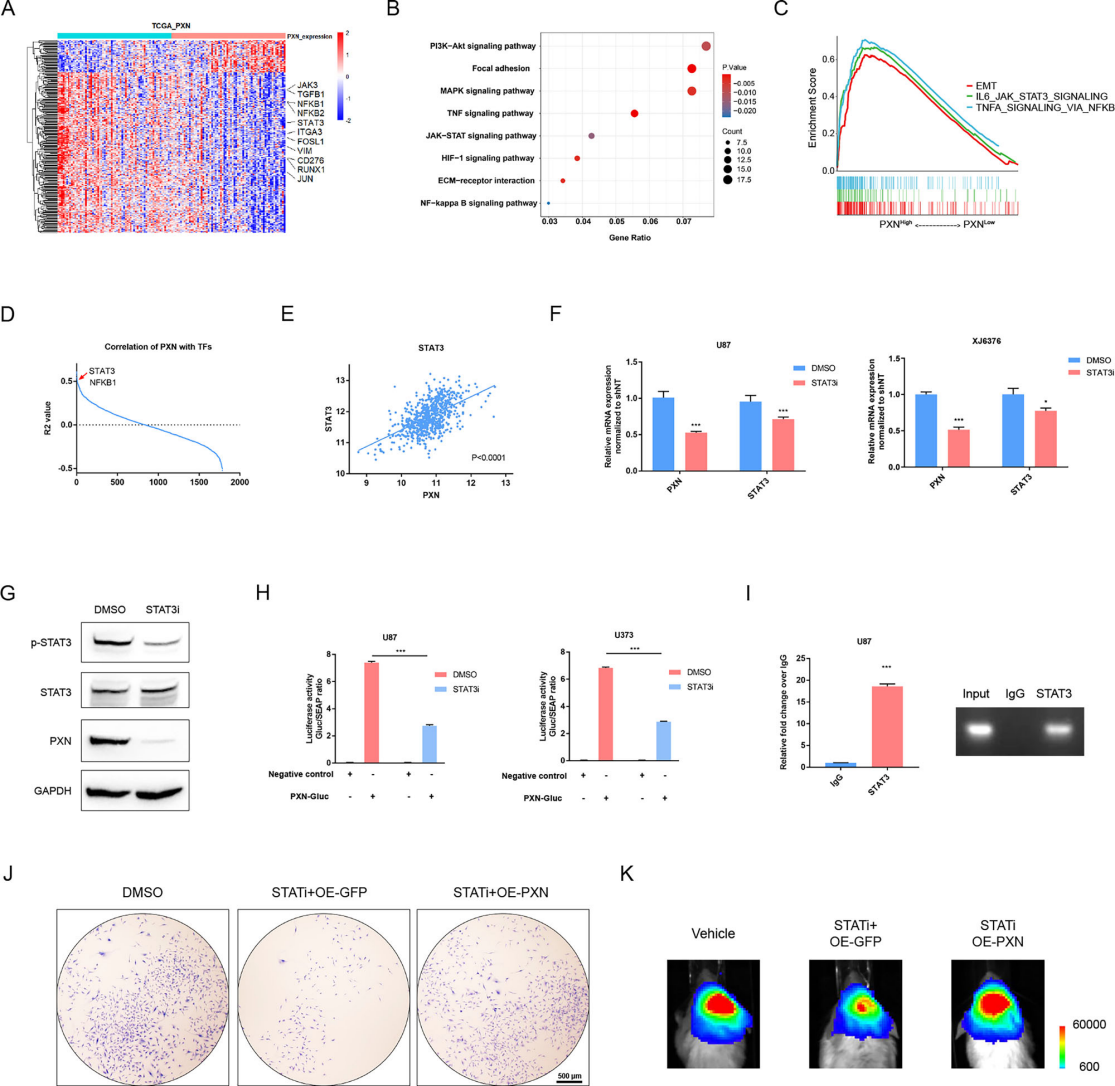
STAT3 acts as an upstream factor of PXN to drive GBM progression. **A** A heatmap showing DEGs between the PXN^High^ and PXN^Low^ groups using information from TCGA dataset. **B** KEGG analysis showing pathways enriched in the PXN^High^ group. **C** GSEA showing oncogenic pathways enriched in the PXN^High^ group. **D** Comparative analysis determining transcription factor (TFs) closely linked with PXN mRNA expression. **E** Correlation analysis between PXN and STAT3 mRNA expression. Pearson’s correlation coefficient (r) was calculated to assess the linear relationship, with statistical significance determined by a two-tailed Student’s *t*-test. **F** qRT-PCR showing PXN mRNA expression in U87 and XJ6376 cells pre-treated with DMSO or STAT3i. Data are presented as mean ± SD of three independent biological replicates. Statistical significance was determined by a two-tailed Student’s *t*-test (*P < 0.05, ***P < 0.001). **G** Western blotting detecting STAT3, p-STAT3 and PXN protein expression in U87 cells pre-treated with DMSO or STAT3i. **H** Luciferase activity assay of U87 and U373 cells co-transfected with PXN-Gluc promoter reporter vector or negative control vector (containing non-promoter sequence) in DMSO and STAT3i-treated groups. Data are presented as mean ± SD of three independent biological replicates. Statistical significance was determined by a two-tailed Student’s *t*-test (***P < 0.001). **I** ChIP-PCR detecting enrichment of STAT3 at PXN promoter in U87 cells, and representative agarose gel electrophoresis images of corresponding ChIP samples amplified using PXN promoter-specific primers. Data are presented as mean ± SD of three independent biological replicates. Statistical significance was determined by a two-tailed Student’s *t*-test (***P < 0.001). **J** Colony formation assay detecting tumorigenic potential of U87 cells subjected to the indicated treatments. Scale bar = 500 μm. **K** Representative BLIs of mice intracranially injected with luciferase-labeled U87 cells subjected to the indicated treatments.

Based on the above results, understanding the underlying mechanisms driving PXN’s role in GBM progression is imperative. Firstly, we conducted bioinformatics analyses leveraging TCGA and CGGA datasets to identify PXN-associated molecular networks. DEGs revealed an enrichment of multiple oncogenes, including STAT3, NFKB1, and VIM, in the PXN^High^ group (Fig. 5A and S3A). Kyoto Encyclopedia of Genes and Genomes (KEGG) pathway analysis uncovered significant enrichment of tumor-related pathways such as TNF signaling, NFκB signaling, and JAK-STAT signaling in the PXN^High^ group (Fig. 5B and S3B), a trend further corroborated by gene set enrichment analysis (GSEA) (Fig. 5C and S3C).

Subsequently, we aimed to identify candidate transcription factors closely linked with PXN. Correlation analysis showed that STAT3 and NFKB1 are top genes positively correlated with PXN (Fig. 5D, E and S3D). In addition, qRT-PCR showed that silencing of STAT3 using lentivirus or STAT3 inhibitor (STAT3i) significantly decreases PXN mRNA expression (Fig. 5F and S3E). Western blotting assays showed that STAT3i decreases the expression of phosphorylated STAT3 (p-STAT3) and PXN (Fig. 5G and S3F). For further validation, a luciferase activity assay showed that inhibition of STAT3 with STAT3i significantly decreases the transcriptional activity of the PXN promoter (Fig. 5H). Additionally, ChIP-PCR confirmed the direct binding of STAT3 at the PXN promoter region (Fig. 5I and S3G).

### PXN acts as a downstream target for STAT3

To explore whether PXN acts as a functional factor for STAT3, we determined to assess the effects of OE-PXN on STAT3i. The results showed that OE-PXN significantly attenuates the inhibitory effects of STAT3i on cell proliferation (Figure S3H). Colony formation and transwell assays indicated that OE-PXN rescued the diminished self-renewal and migration of GBM cells after STAT3i treatment (Fig. 5J and S3I). Next, K-M analysis demonstrated that mice in the OE-PXN group suffer a much shorter survival time than the OE-GFP group (Fig. S3J). BLI analysis further confirmed the higher tumorigenic ability of PXN-overexpression cells (Fig. 5K). These data indicate that PXN is a functional downstream effector of STAT3.

### PXN feedback activates STAT3 via regulating SRC transcriptional expression

**Fig. 6:**
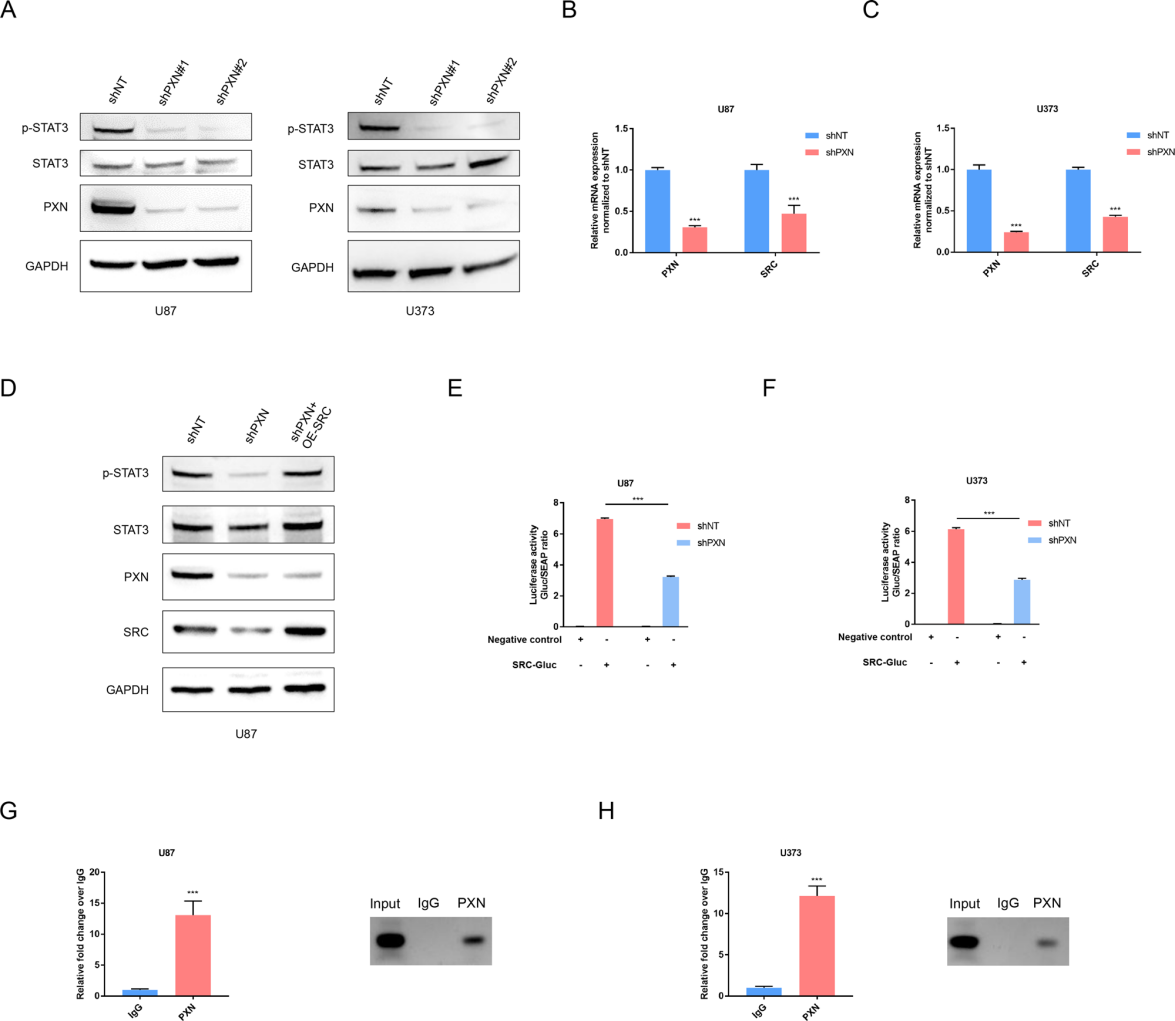
PXN feedback activates the STAT3 pathway by regulating SRC transcriptional expression. **A** Western blotting determining p-STAT3, STAT3 and PXN protein expression in U87 and U373 cells transfected with NT-shRNA and PXN-shRNA. **B**, **C** qRT-PCR detecting SRC mRNA expression in U87 and U373 cells transfected with NT-shRNA and PXN-shRNA. Data are presented as mean ± SD of three independent biological replicates. Statistical significance was determined by a two-tailed Student’s *t*-test (***P < 0.001). **D** Western blotting determining p-STAT3 and STAT3 protein expression in U87 cells subjected to the indicated treatments. **E**, **F** Luciferase activity assay of U87 and U373 cells co-transfected with SRC-Gluc promoter reporter vector or negative control vector (containing a non-promoter sequence) in NT-shRNA-transfected and PXN-shRNA-transfected groups. Data are presented as mean ± SD of three independent biological replicates. Statistical significance was determined by a two-tailed Student’s *t*-test (***P < 0.001). **G**, **H** ChIP-PCR showing enrichment of PXN at SRC promoter in U87 and U373 cells, and representative agarose gel electrophoresis images of corresponding ChIP samples amplified using SRC promoter-specific primers. Data are presented as mean ± SD of three independent biological replicates. Statistical significance was determined by a two-tailed Student’s *t*-test (***P < 0.001).

Interestingly, western blotting assays showed a significant decrease in p-STAT3 protein levels upon PXN silencing, while the total STAT3 protein remained unchanged (Fig. 6A). Thus, we examined potential interactions between PXN and STAT3-associated kinases, but no obvious interactions were detected (Supplementary table 1). Evidence indicated that PXN exerts its nuclear function to transcriptionally regulate SRC, a well-known activation factor of STAT3 [26]. Consistently, treatment with an SRC inhibitor significantly reduced p-STAT3 levels in GBM (Fig. S4A). In parallel, western blot analysis of cytoplasmic and nuclear fractions confirmed the nuclear presence of PXN (Fig. S4B). Moreover, PXN nuclear translocation was critically dependent on its phosphorylation status (Fig. S4C, D). Based on these results, we performed qRT-PCR to detect the mRNA expression of SRC after PXN silencing, revealing a significant reduction (Fig. 6B, C). Western blotting showed that PXN knockdown leads to a pronounced reduction in SRC and p-STAT3 levels, whereas SRC overexpression rescued p-STAT3 expression (Fig. 6D). Collectively, these results indicate that PXN may activate STAT3 signaling through transcriptional regulation of SRC. To further validate this mechanism, a luciferase activity assay was performed, showing that silencing of PXN significantly decreases the transcriptional activity of the SRC promoter (Fig. 6E, F). ChIP-PCR analysis further revealed a significant binding of PXN to the SRC promoter region (Fig. 6G, H). These findings underscore the critical role of PXN-mediated transcriptional regulation of SRC in activating STAT3, and, together with our previous results, support its involvement in a potential feedback mechanism.

### PXN enhances YB-1 stability by inhibiting ubiquitin-mediated degradation

**Fig. 7:**
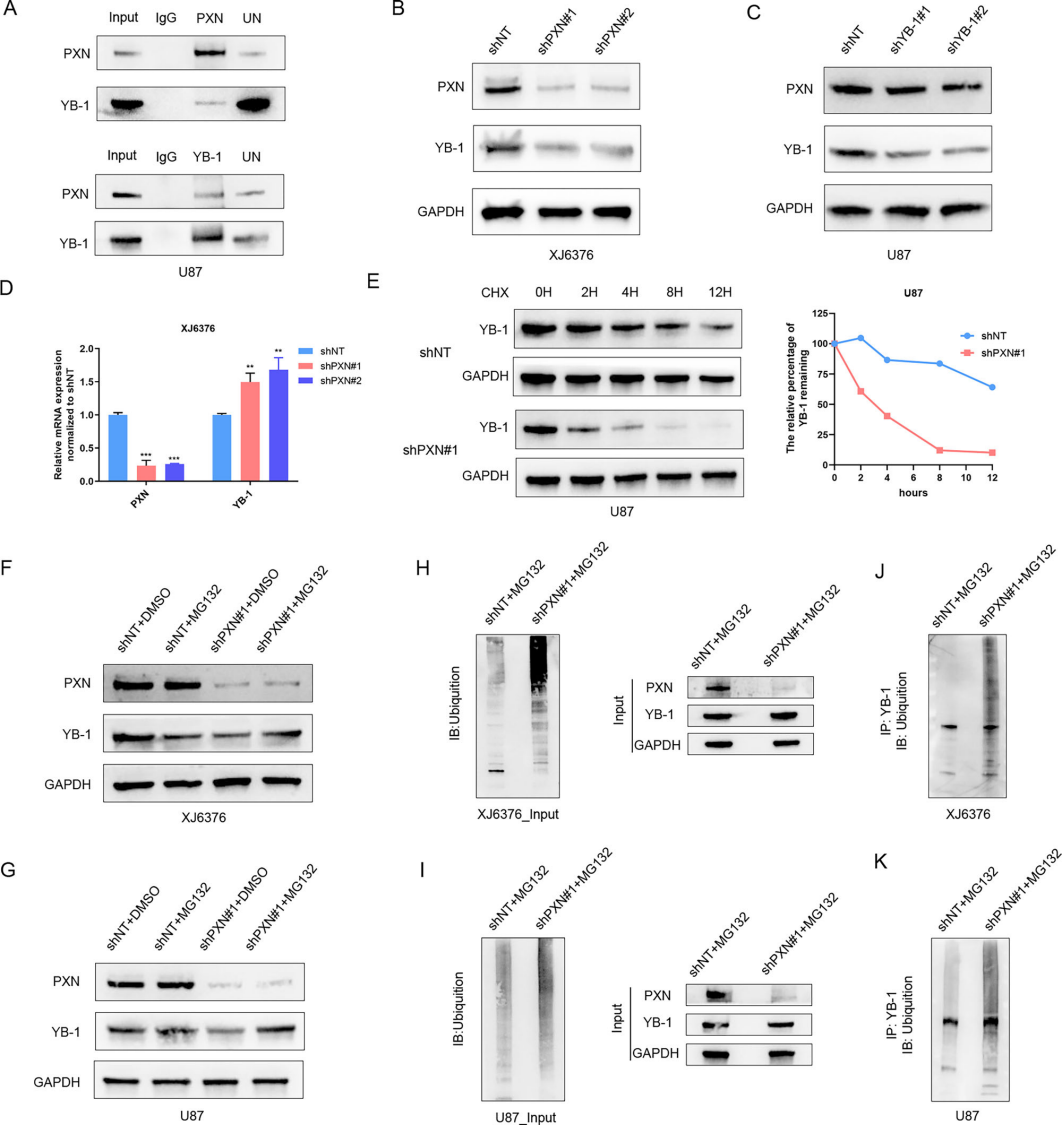
PXN enhances YB-1 stability by inhibiting ubiquitin-mediated degradation. **A** Co-IP assays validating the binding relationship between PXN and YB-1 proteins. **B** Western blotting determining YB-1 protein expression in XJ6376 cells transfected with NT-shRNA or PXN-shRNA. **C** Western blotting determining PXN protein expression in U87 cells transfected with NT-shRNA or YB-1-shRNA. **D** qRT-PCR detecting YB-1 mRNA expression in XJ6376 cells transfected with NT-shRNA and PXN-shRNA. Data are presented as mean ± SD of three independent biological replicates. Statistical significance was determined by a one-way ANOVA with Dunnett’s multiple comparisons test (**P < 0.01, ***P < 0.001). **E** Western blotting comparing YB-1 protein degradation rate in U87 cells transfected with NT-shRNA and PXN-shRNA. **F**, **G** Western blotting determining YB-1 protein alterations in XJ6376 (**F**) and U87 (**G**) cells subjected to the indicated treatments. **H**, **I** Western blotting determining ubiquitin level in XJ6376 (**H**) and U87 (**I**) cells transfected with NT-shRNA and PXN-shRNA using input samples. **J**, **K** Western blotting determining ubiquitin level in XJ6376 (**J**) and U87 (**K**) cells transfected with NT-shRNA and PXN-shRNA, followed by immunoprecipitation with a YB-1 antibody.

The identified proteins through mass spectrometry analysis (Supplementary table 1) were subjected to bioinformatics analysis using DAVID website (https://david.ncifcrf.gov/). For the molecular function (MF) annotation from Gene Ontology (GO), the top 20 results were visualized using a bubble plot (Fig. S5A). The results indicate that proteins interacting with PXN are strongly linked with mRNA binding and DNA binding. Among these identified proteins, YB-1 is recognized for its ability to bind both DNA and RNA [27]. We therefore investigated the interaction between PXN and YB-1. Co-IP experiments confirmed the binding between PXN and YB-1 (Fig. 7A). To explore the regulatory relationship between PXN and YB-1, shRNAs targeting PXN or YB-1 were introduced into GBM cells. The results showed that silencing of PXN significantly reduces YB-1 protein expression (Fig. 7B and S5B). However, silencing YB-1 did not lead to a decrease in PXN protein level (Fig. 7C and S5C). Meanwhile, qRT-PCR results showed that YB-1 mRNA has no significant reduction after silencing PXN (Fig. 7D and S5D). These results imply that PXN’s regulation of YB-1 likely occurs through post-translational modification (PTM). To further verify this, the protein synthesis inhibitor cycloheximide (CHX, MCE, HY-12320) was utilized. Western blotting assays showed that silencing PXN accelerates YB-1 protein degradation (Fig. 7E and S5E), indicating that PXN contributes to YB-1 stability. Given that the ubiquitin-proteasome system (UPS) is a primary pathway for protein degradation [28], the proteasome inhibitor MG132 (MCE, HY-13259) was introduced to the study. Western blotting analysis indicated that MG132 treatment can reverse the reduction of YB-1 protein after PXN knockdown (Fig. 7F, G), indicating PXN enhances YB-1 stability, likely by inhibiting ubiquitin-mediated degradation. Further experiments showed that silencing PXN increases overall cellular ubiquitin levels in GBM cells (Fig. 7H, I). Meanwhile, the protein complex co-precipitated by the anti-YB-1 antibody also exhibited increased ubiquitin levels (Fig. 7J, K). Collectively, these findings indicate that PXN enhances YB-1 stability by inhibiting ubiquitin-mediated degradation.

### YB-1 sustains the malignant features of GBM by regulating multiple signaling pathways

**Fig. 8:**
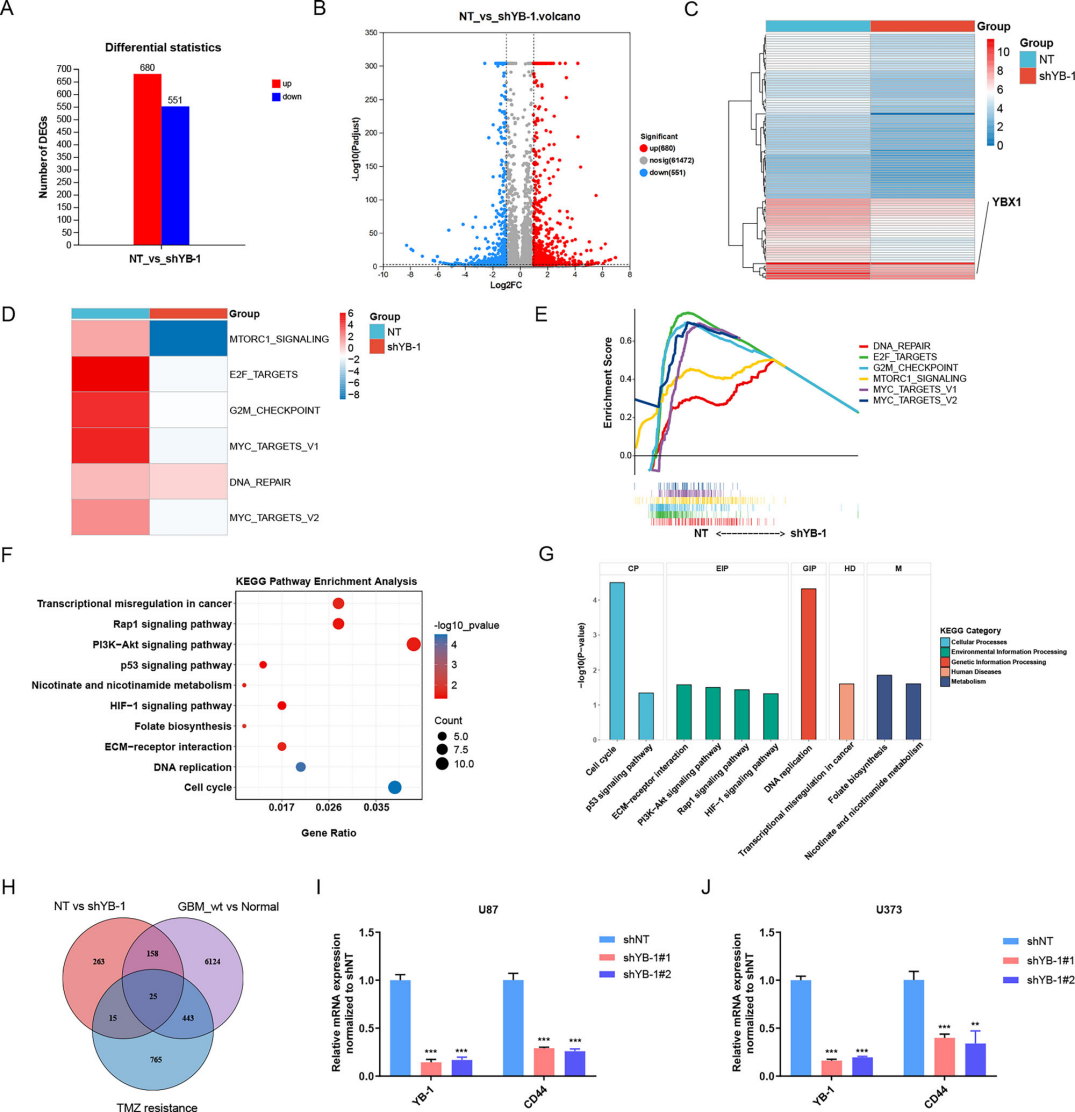
YB-1 sustains the malignant features of GBM by regulating multiple signaling pathways. **A** A column chart showing the number of DEGs between shNT and shYB-1 groups. **B** A volcano plot showing DEGs between shNT and shYB-1 groups. **C** A heatmap displaying mRNA expression profiles of top 200 genes between shNT and shYB-1 groups. **D** Heatmap showing enrichment differences of HALLMARK gene sets between NT and shYB-1 groups, based on GSEA calculated from logFC data. **E** Enrichment plot of the HALLMARK gene sets in NT vs shYB-1 groups, generated from the official GSEA website. **F** KEGG analysis of the down-regulated genes showing enrichment in oncogenic pathways. **G** KEGG analysis of the down-regulated genes showing the categories of enriched oncogenic pathways. **H** A Venn plot showing down-regulated genes in shYB-1 group linked with TMZ-resistance. **I**, **J** qRT-PCR detecting CD44 mRNA expression in U87 and U373 cells transfected with NT-shRNA and YB-1-shRNA. Data are presented as mean ± SD of three independent biological replicates. Statistical significance was determined by a one-way ANOVA with Dunnett’s multiple comparisons test (**P < 0.01, ***P < 0.001).

The role of YB-1 in promoting GBM malignant features has been widely recognized [29, 30]. A study has identified a YB-1/CCT4/mLST8/mTOR pathway that promotes GBM growth [30]. However, the comprehensive regulatory landscape of YB-1 in GBM cells remains unclear. Thus, mRNA sequencing was conducted to compare non-targeting (NT)-shRNA and YB-1-shRNA transfected U87 and U373 cells, identifying a total of 680 up-regulated and 551 down-regulated genes (Fig. 8A, B). The top 100 DEGs were visualized in a heatmap (Fig. 8C). GSEA analysis revealed significant pathway alterations at a whole gene level, indicating that YB-1 silencing down-regulates multiple oncogenic pathways, including MTORC1 signaling, E2F targets, and G2M checkpoint (Fig. 8D, E). Further KEGG analysis of the down-regulated genes showed enrichment in oncogenic pathways (Fig. 8F, G). Given the above findings linking PXN with TMZ resistance (Fig. 4 and Fig. S2), we focused on identifying key down-regulated genes associated with TMZ resistance. First, TMZ resistance-associated genes were obtained from GeneCards (https://www.genecards.org/). DEGs between GBM, IDH-wildtype and normal samples were identified to determine tumor-specific genes. By intersecting these with down-regulated genes identified by mRNA sequencing, a total of 25 candidate genes potentially linked to TMZ resistance were obtained (Fig. 8H). Among them, CD44 is widely known to promote TMZ resistance [31], prompting us to verify the relationship between YB-1 and CD44 using qRT-PCR, which showed that YB-1 silencing leads to decreased CD44 mRNA expression (Fig. 8I, J). In conclusion, YB-1 promotes GBM malignancy by influencing multiple oncogenic genes and pathways.

## Discussion

GBM is the most malignant primary brain tumor in adults. Despite extensive investigations into targeted or tailored therapies, patient survival has yet to improve significantly [32]. These failures have intensified the search for novel therapeutic approaches for GBM. According to the WHO 2021 classification, GBM, IDH-wildtype is defined by the presence of one or more of the following molecular features: epidermal growth factor receptor (EGFR) amplification, TERT promoter mutation, and the combined gain of chromosome 7 and loss of chromosome 10 [2]. The median overall survival (OS) of patients with GBM, IDH-wildtype ranges from 12 to 21 months, with only 7% of patients surviving five years post-diagnosis [4]. Given the dismal prognosis and limited therapeutic options, we explored prognosis-related genes in GBM, IDH-wildtype, and identified PXN as a key player.

Previous studies have preliminarily explored PXN’s role in GBM [24, 25], confirming its basal mRNA and protein expression, as well as its prognostic significance. However, functional validation of PXN in these studies was restricted to self-renewal, migration, and invasion assays. Our investigation provides a more comprehensive understanding of its involvement in GBM, IDH-wildtype progression, including in vitro proliferation, migration, self-renewal, therapeutic response, and in vivo tumorigenic capacity, through PXN silencing and overexpression in multiple cell lines. The evidence indicated that EGFR amplification is a crucial feature of GBM, IDH-wildtype, with amplified EGFR driving GBM progression by activating the JAK/STAT3 pathway [33]. Using bioinformatic analysis, we found that PXN mRNA levels were positively correlated with STAT3 mRNA expression. A previous study has indicated that PXN promoter region contains putative STAT3 binding sites [11]. Building on this, we experimentally demonstrated that STAT3 directly promotes PXN transcription by binding to its promoter. These results provide further insights into the molecular mechanisms regulating PXN expression in GBM.

The role of PXN in facilitating GBM progression is widely recognized. However, most studies have focused on its FAs or scaffold function [18–23]. Thus, little is known about its nuclear actions. In this study, we observed an unexpected result: silencing PXN led to a decrease in p-STAT3 levels, rather than total STAT3. We firstly hypothesized that PXN may form a complex with STAT3 or its upstream kinases, such as JAK, SRC, and EGFR [34]. However, mass spectrometry analysis did not support our hypothesis. A previous study showed that phosphorylated PXN can translocate into the nucleus, transcriptionally promoting AR and ERK expression in prostate cancer [12]. Additionally, researchers proposed a novel mechanism whereby nuclear PXN promotes SRC transcription to enhance ovarian cancer angiogenesis [26]. Based on these findings, we investigated PXN nuclear localization, and found that PXN localizes to the nucleus, particularly in a phosphorylation-dependent manner. Furthermore, our experimental results demonstrated PXN directly binds to the SRC promoter, transcriptionally activating its expression. SRC, a non-receptor protein tyrosine kinase, is pivotal in regulating signal transduction by various cell surface receptors in a wide range of cellular events [35, 36]. STAT3 phosphorylation is dependent on SRC kinase activity [34]. By integrating published studies with our results, we validated that PXN directly promotes SRC transcription, leading to increased STAT3 phosphorylation. Together with our above findings that STAT3 directly promotes PXN transcription, these results suggest a positive feedback loop of the STAT3-PXN axis in GBM.

Pathway analyses of PXN-associated proteins identified by mass spectrometry revealed enrichment in mRNA binding and DNA binding functions. Among these proteins, YB-1, an RNA and DNA binding protein, plays multiple roles in gene transcription, RNA splicing, DNA damage repair, cell cycle, and immunity across various cancers [27]. A study demonstrated that YB-1/CCT4/mLST8/mTOR forms a positive feedback loop to promote GBM progression [30]. Besides mTOR, non-coding RNA may also indirectly regulate YB-1 [37]. However, the upstream regulatory factors of YB-1, particularly at the protein level, remain unclear. Following mass spectrometry analysis and Co-IP, we found a binding relationship between PXN and YB-1 proteins. Further experiments demonstrated that PXN regulates YB-1 stability, rather than YB-1 regulating PXN. Mechanistically, PXN stabilizes YB-1 by inhibiting its ubiquitin-mediated degradation. A study showed that PXN enhances NLRP3 deubiquitination by recruiting deubiquitinating enzymes USP13 [38]. However, identified proteins by mass spectrometry analysis in our study do not include recognized deubiquitinating enzymes. Therefore, we speculate that PXN inhibits YB-1 ubiquitination through additional mechanisms, such as physically shielding ubiquitination sites, recruiting low-abundance deubiquitinating enzymes, or altering YB-1 conformation. To our knowledge, this is the first evidence that PXN stabilizes YB-1 by modulating its post-translational modifications, although the detailed mechanisms require further investigation.

Although YB-1 has been studied in glioma, its transcriptional profiles in GBM cells are incompletely characterized. Thus, we transfected U87 and U373 cells with NT-shRNA and YB-1-shRNA, and performed mRNA sequencing. CCT4 mRNA levels showed no significant change in our sequencing data, as experimentally validated in the previous study [30]. The same study also reported that YB-1 correlates with activated mTOR signaling, consistent with our GSEA analysis. Among the DEGs identified in mRNA sequencing, CD44 is a candidate downstream gene associated with YB-1. Given the well-documented role of CD44 in TMZ resistance [31], this association suggests that YB-1 may mediate TMZ resistance via CD44. This finding is complementary to previously reported YB-1-mediated TMZ resistance via the MDM2/p53 pathway [39]. Overall, we analyzed global gene and pathway alterations associated with YB-1 silencing through mRNA sequencing, providing insights into its potential molecular mechanisms underlying TMZ resistance in GBM.

These findings indicate the pleiotropic role of PXN as both a signal amplifier and a multi-pathway integrator in GBM malignancy. Importantly, PXN expression positively correlates with poor prognosis, suggesting a highly promising therapeutic target. In addition, our study identifies multiple potential targets within the regulatory network, indicating that combination therapies may provide synergistic benefits. Nevertheless, conventional targeted therapies often have systemic side effects, as many cancer-related targets are involved in normal physiological processes. Common adverse effects include asthenia, gastrointestinal disturbances, skin reactions, and systemic complications such as cardiotoxicity and hypertension [40]. Additionally, off-target effects may further limit therapeutic efficacy [41]. Moreover, effective drug delivery to brain tumors is limited by the blood-brain barrier [42]. To address these challenges, dual- and multi-targeted nanoparticles that integrate multiple targeting functionalities have emerged as a promising strategy for precise drug delivery to GBM [43]. Taken together, these findings highlight several targets for the development of novel GBM treatments.

Collectively, our results provide a comprehensive analysis of PXN’s role in GBM, reinforcing its significance as a GBM biomarker. At the upstream level, PXN is transcriptionally regulated by STAT3, and this study for the first time suggests a STAT3-PXN-SRC-STAT3 positive feedback loop in GBM. Downstream, PXN plays a key role in stabilizing YB-1 protein through inhibition of its ubiquitin-mediated degradation. Together, these findings define a molecular network that may serve as a therapeutic target in GBM.

## Materials and methods

### Bioinformatics analysis

The detailed information regarding datasets and bioinformatics analysis can be found in the Supplementary materials.

### Lentivirus production and transduction

The detailed process for cell cultures, lentivirus production and transduction can be found in the Supplementary materials. The target sequences for the short hairpin RNAs (shRNAs) used in this study are as follows:

shPXN#1: GCCTTACTGTCAGAACTGCTT;

shPXN#2: CCCAACTGGAAACCACACATA;

shSTAT3: GCACAATCTACGAAGAATCAA;

shYB-1#1: AGCAGACCGTAACCATTATAG;

shYB-1#2: CCAGTTCAAGGCAGTAAATAT;

The PXN-overexpression lentivirus was designed and synthesized by Sigma-Aldrich, catalog number: TRCN0000123137.

### Functional assays

The detailed process for cell viability, colony formation, wound healing and transwell assays can be found in the Supplementary materials.

### RNA isolation and quantitative real-time polymerase chain reaction (qRT-PCR)

The detailed protocols can be found in the Supplementary materials. The primer sequences applied in the study are as follows:

- PXN (forward CCACCACCTTCTAAAACGTCAG; reverse CCCAAGCATTGAGTCCAGGG)
- STAT3 (forward CAGCAGCTTGACACACGGTA; reverse AAACACCAAAGTGGCATGTGA)
- SRC (forward GTGGACACTCAGGAGAAGAACG; reverse TGCTGCTTAATAATCTTGCCCTT)
- YB-1 (forward GGGGACAAGAAGGTCATCGC; reverse CGAAGGTACTTCCTGGGGTTA)
- CD44 (forward CTGCCGCTTTGCAGGTGTA; reverse CATTGTGGGCAAGGTGCTATT)
- GAPDH (forward TGTGGGCATCAATGGATTTGG; reverse ACACCATGTATTCCGGGTCAAT)

### Western blotting assay

The detailed protocols can be found in the Supplementary materials. Antibodies used in the study are listed below:

- Anti-PXN primary antibody was purchased from Santa Cruz (cat. no. sc-365379).
- Anti-p-STAT3 (cat. no. sc-8059) and Anti-STAT3 (cat. no. sc-8019) primary antibody were purchased from Santa Cruz.
- Anti-SRC primary antibody was purchased from Santa Cruz (cat. no. sc-130124).
- Anti-YB-1 primary antibody was purchased from Santa Cruz (cat. no. sc-101198).
- Anti-Ubiquitin primary antibody (P4D1) was purchased from Santa Cruz (cat. no. sc-8017).
- Anti-GAPDH primary antibody was purchased from CST (cat. no. #3683).
- Anti-beta Actin antibody was purchased from abcam (cat. no. ab115777).
- Anti-Rabbit-IgG (cat. no. #7074) and Anti-Mouse-IgG (cat. no. #7076) were purchased from CST.
- Mouse (cat. no. A00001) and Rabbit IgG (cat. no. A00002) Isotype Control were purchased from ZEN-BIOSCIENCE.
- The full uncropped Western blotting images are provided in the Supplemental Material.

### Flow cytometry assay

The detailed information can be found in the Supplementary materials.

### Intracranial xenograft tumor models

The ethical approval for animal experiments conducted in this study was granted by the Scientific Ethics Committee of the First Affiliated Hospital of Xi’an Jiaotong University, Xi’an, China. Severe combined immunodeficiency (SCID) mice aged 6-8 weeks were used to construct the xenograft model. Mice were randomly divided into 2-3 groups and five mice per group to ensure the stability of the results. Investigators were not blinded to group assignments during data collection. After anesthesia with isoflurane, a GBM cell suspension (1 × 10^5^ cells/5 μL PBS) was administered into the right caudate nucleus. The injection site was determined relative to bregma using the following coordinates: anterior–posterior (AP), +1.0 mm; medial–lateral (ML), +2.5 mm; dorsal–ventral (DV), -3.0 mm from the brain surface. Due to limited cranial space and the injection site being in the functional area, intracranial xenograft GBM tumors can initially cause motor dysfunction in mice during tumor growth process. Mice were sacrificed as motor dysfunction was developed. Thus, the maximum tumor size/burden is not applicable to this study. All authors confirmed that all experiments were performed in accordance with the approved protocol and other relevant guidelines and regulations.

### Immunohistochemistry (IHC)

The detailed process can be found in the Supplementary materials.

### Detection of Caspase-3 activity and Annexin V apoptosis in living cells

The detailed process can be found in the Supplementary materials.

### Chromatin immunoprecipitation (ChIP)

The detailed protocols can be found in the Supplementary materials. The primer sequences for ChIP are as follows:

- PXN (forward GACTGAACATGGTGGGAGCA; reverse ACAGCTTCGAGTTCCCCTTG)
- SRC (forward AGCGTGTCACTGGGTAAATG; reverse GCCCTCTTCCATTCCTTAGAC).

### PXN and SRC promoter luciferase assay

The detailed protocols can be found in the Supplementary materials.

### Co-immunoprecipitation (Co-IP) and Mass spectrometry analysis

The detailed protocols can be found in the Supplementary materials.

### mRNA sequencing analysis

The detailed protocols can be found in the Supplementary materials.

### Immunofluorescence

The detailed protocols can be found in the Supplementary materials. The antibodies used included:

- Phospho-Paxillin (Tyr118) Recombinant monoclonal antibody (proteintech, Cat No. 81180-2-RR, Rabbit / IgG)
- Goat anti-Rabbit IgG (H + L) Highly Cross-Adsorbed Secondary Antibody, Alexa Fluor™ Plus 488 (Thermo Fisher Scientific, Cat No. A32731).

### Nuclear-cytoplasmic fractionation

Nuclear and cytoplasmic fractions were prepared from GBM cells using the Nuclear and Cytoplasmic Extraction Kit (Beyotime, P0027) according to the manufacturer’s instructions.

### Statistical analysis

All data are presented as mean ± standard deviation (SD). Statistical differences between the two groups were evaluated by a two-tailed *t*-test. Comparison among multiple groups was performed using one-way analysis of variance (ANOVA) followed by Tukey’s post hoc test or Dunnett’s multiple comparisons test. Pearson’s correlation coefficient (r) was calculated to assess the linear relationship. The significance of the Kaplan-Meier (K-M) analysis was determined by log-rank test. Statistical analysis was performed using SPSS 22.0 or GraphPad Prism 6. A p-value < 0.05 was considered as statistically significant.

Supplementary information is available at Cell Death Discovery’s website.

## Supplementary information

Supplementary table 1

Supplementary Material

Original western blots

## Data Availability

The data used in this study can be downloaded from TCGA dataset (https://xenabrowser.net/datapages/) and the CGGA dataset (http://www.cgga.org.cn). The mRNA sequencing data have been deposited in the Gene Expression Omnibus (GEO) under accession number GSE294052. The R code used in this study is available from the corresponding author upon reasonable request.
