# Supplementary material for: Deciphering the STAT3-PXN positive feedback loop in GBM, IDH-wildtype: transcriptional regulation and inhibition of YB-1 ubiquitination
Source: Cell Death Discov. 2026 Mar 23;12:168. doi: 10.1038/s41420-026-03035-9 (PMC13039979; doi:10.1038/s41420-026-03035-9)
Supplement: Supplementary file 2 — Supplementary Material [file 41420_2026_3035_MOESM2_ESM.docx]

**Supplementary materials and methods**

**Patient inclusion and datasets**

In total, 205 GBM, isocitrate dehydrogenase (IDH)-wildtype samples and 5 normal brain samples were used in the current study. 132 GBM, IDH-wildtype samples and 5 normal brain samples were obtained from the UCSC Xena platform^[1]^, which aggregates TCGA RNA sequencing (RNA-seq) dataset^[2, 3]^. 73 GBM, IDH-wildtype samples were derived from CGGA RNA-seq dataset^[4, 5]^. Detailed data processes can be found in the previous study^[6]^. The corresponding clinical information was obtained from TCGA (<https://portal.gdc.cancer.gov/>) and CGGA (<http://www.cgga.org.cn>) datasets.

**Bioinformatics analysis**

Univariate Cox proportional hazards regression analysis was conducted to identify the core prognosis-related genes in GBM, IDH-wildtype. Differential expression analysis was used to identify highly expressed tumor-specific genes. The core gene of this study was determined through comparative analysis. The detailed protocols for Kyoto Encyclopedia of Genes and Genomes (KEGG) and gene set enrichment analysis (GSEA) are available in the prior study^[6]^.

**Cell cultures**

The following major reagents were used for cell culture: DMEM-F12 (Gibco), fetal bovine serum (FBS, NEWZERUM), penicillin-streptomycin (MCE), and trypsin-EDTA (Gibco). The U87, U251, U373, HEK 293T, and normal human astrocytes (NHA) cell lines were provided by the First Affiliated Hospital of Xi'an Jiaotong University. The cells were cultured in DMEM supplemented with 10% FBS and 1% penicillin-streptomycin. The medium was changed every 3-4 days.

Primary cells (XJ6376) derived from a GBM, IDH-wildtype patient were obtained from surgical samples in the Department of Neurosurgery, the First Affiliated Hospital of Xi'an Jiaotong University. Informed consent was obtained from the subject. Fresh surgical GBM specimens were collected within 30 minutes after tumor isolation, and rinsed in phosphate-buffered saline (PBS) to remove adherent blood and visible necrotic tissue. Tissues were divided equally into three parts for RNA extraction, protein extraction, and cell culture. For cell culture, mechanical and trypsin-EDTA dissociation were conducted to obtain single-cell suspensions. Single cells were seeded in DMEM containing 10% FBS and 1% antibiotics, and incubated under sterile conditions with 5% CO_2_ at 37℃.

All cell lines used in this study were analyzed by short tandem repeat typing and mycoplasma serology, with the most recent assessment conducted in September 2022. Cells were passaged 2-6 times for experimental purposes and revived every 3-4 months.

**Lentivirus production and transduction**

Plasmid DNA was extracted using a mini plasmid purification kit ([TIANGEN](https://www.tiangen.com/)). DNA concentration was measured by Nanodrop 2000 (Thermo Fisher). HEK293T cells were cultured until reaching ~70-80% confluence, then the medium was replaced with antibiotic-free medium containing 10% FBS for transfection. Next, they were transfected with the pLKO.1-TRC cloning vectors (Addgene) together with the packaging plasmids psPAX2 (Addgene) and pMD2.G (Addgene), using Calcium Phosphate Cell Transfection Kit ([Beyotime](https://beyotime.com/news.do?method=newsframe1&pageurl=about-beyotime.htm)). After 12 hours, the culture medium was changed to a complete medium. Viral supernatants were collected 24 hours and 48 hours later. PEG-8000 ([Beyotime](https://beyotime.com/news.do?method=newsframe1&pageurl=about-beyotime.htm)) was used to precipitate the lentiviruses. GBM cells were treated with medium containing lentiviruses for 16 hours in the presence of 8 µg/mL polybrene. Afterwards, the medium was changed, and the cells were cultured for 3-4 days.

**Cell viability assay**

The cell number was determined using an automated cell counter. Then cells were seeded into a 96-well plate at 1*10^3^ cells/well density in triplicate, and cultured in a complete medium with 5% CO_2_ at 37℃. After the indicated periods, CCK8 reagent (GlpBio) was added into each well, and absorbance was measured 4 hours later using Synergy HTX multi-mode reader (BioTek).

**Colony formation assay**

The colony formation assay was conducted to detect the *in vitro* tumorigenic potential of GBM cells. GBM cells were seeded into 6-well plates at 2×10^3^ cells/well. After 14 days of cultivation in a complete medium, the cells were fixed with methanol and stained with methylene blue. The number of clones was counted to determine the self-renewal ability of GBM cells.

**Wound healing assay**

The wound healing assay was applied to evaluate the migratory ability of GBM cells. GBM cells were seeded into 6-well plates at 3×10^5^ cells/well and cultured in a complete medium. Once cells reached approximately 90% confluence, linear wounds were generated using sterile pipette tips. Afterwards, the cells were washed 3 times with PBS to remove detached cells. Images were taken using an inverted microscope. The leading edges were delineated by black lines.

**Transwell assay**

The transwell assay was performed to assess the migration ability of GBM cells. 600 µL medium containing 10% FBS was added into the lower chamber (Corning Falcon). GBM cells were seeded into the upper chamber containing 200 µL of serum-free medium, at a density of 2×10^4^ cells/well. After approximately 16 hours, GBM cells were fixed with methanol and stained with methylene blue. Subsequently, the non-migrated cells were gently removed using cotton swabs, and images of migrated cells were taken under an inverted microscope.

**RNA isolation and quantitative real-time polymerase chain reaction (qRT-PCR)**

Total RNA was prepared by TRIzol reagent (Beyotime) following the manufacturer’s protocol. RNA concentration was measured by Nanodrop 2000. cDNA was synthesized using RevertAid^TM^ Master Mix, with DNase I (Thermo MBI) according to the manufacturer’s instructions. qRT-PCR was carried out using the StepOnePlus real-time PCR system with SYBR Green Mix (DIYIBIO). GAPDH was used as an internal control. Running cycles for DNA amplification are were as follows: 95 °C (30 s), 40 cycles of 95 °C (10 s), 60 °C (10 s), and 72 °C (30 s). Relative mRNA expression was calculated using 2^-ΔΔCT^ method.

**Western blotting assay**

GBM cells were homogenized on ice in RIPA buffer containing 1% protease and phosphatase inhibitor cocktail (MCE), and then centrifuged at 14,000 × g for 10 min to acquire protein supernatants. Protein concentration was determined using the bicinchoninic acid (BCA) assay kit (Beyotime). Equal amounts of protein lysates (20 μg/lane) were subjected to SDS-PAGE FuturePAGE^TM^ 4%-12% gels and transferred onto a polyvinylidene fluoride (PVDF) membrane (Thermo Scientific). The membrane was blocked with 5% non-fat milk (Blotting Grade Blocker, Beyotime) for 1 hour at room temperature, and then incubated with the corresponding primary antibody overnight at 4℃. The next day, the membranes were incubated with peroxidase-conjugated secondary antibodies for 1 h at room temperature. Then, the signals were visualized using the Amersham enhanced chemiluminescence (ECL) Western Blotting System.

**Flow cytometry assay**

GBM cells were stained with an Alexa Fluor^®^ Annexin V/Dead Cell Apoptosis Kit (Thermo Scientific), strictly following the manufacturer’s protocol. After staining, cell apoptosis was assessed by Attune NxT Flow Cytometer (Thermo Scientific).

**Bioluminescent imaging (BLI)**

GBM cells were infected with lentiviral particles (pHAGE PGK-GFP-IRES-LUC-W) to co-express luciferase and GFP. GFP-expressing cells were sorted by fluorescence-activated cell sorting (FACS) (BD Biosciences) and subsequently implanted into mice to establish xenograft models. For in vivo imaging, mice were injected intraperitoneally with XenoLight D-luciferin (2.5 mg/100 µL, PerkinElmer). Then, mice were anesthetized with isoflurane for image capture using an IVIS 100 imaging system (PerkinElmer).

**Immunohistochemistry staining (IHC)**

Paraffin-embedded tumor tissue sections were incubated with corresponding primary antibodies (Anti-PXN primary antibody, cat. no. sc-365379) at 4℃ overnight. The next day, the slices were incubated for 1 hour with HRP-conjugated secondary antibodies at room temperature and visualized using 3,3'-diaminobenzidine (DAB) substrate solution. Images were taken by a microscope (Servicebio Technology Co., Ltd., Wuhan, China).

**Detection of caspase-3 activity and Annexin V apoptosis in living cells**

XJ6376 primary GBM cells were seeded into 24-well plates. When cells reached 70–80% confluence, a mixture containing 194 μL of Annexin V-mCherry Binding Buffer, 5 μL of Annexin V-mCherry, and 1 μL of GreenNuc™ Caspase-3 Substrate from the Beyotime kit (C1077S) was added to each well. After 20-30 minutes of light-avoidance incubation at room temperature, images were taken under an inverted microscope.

**Chromatin immunoprecipitation (ChIP)**

ChIP was carried out strictly following the manufacturer's instructions. Bioruptor UCD-200 was applied for DNA sonication, and 3*10^6^ cells were used per reaction. After centrifugation at 10,000×g for 10 minutes, 10% of the supernatant was reserved as input. The remaining supernatant was subjected to immunoprecipitation with either the corresponding primary antibody or normal IgG as a negative control, followed by incubation with Protein G Dynabeads. The immunoprecipitated DNA was purified and subsequently analyzed by quantitative PCR (qPCR) and agarose gel electrophoresis to verify the enrichment of target sequences.

**Luciferase reporter assay for PXN and SRC promoter activity**

The PXN promoter region (−1811 to −424 from TSS) and SRC promoter region (−1770 to −882 from TSS) were amplified from human genomic DNA. U87 and U373 GBM cells were seeded in 24-well plates for 24 h. When cells reached 50–70% confluence, they were co-transfected with PXN or SRC promoter reporter plasmids along with a Renilla luciferase plasmid using lipofectamine 3000 (Thermo Scientific, L3000001) according to the manufacturer’s protocol. After 48 hours, promoter activity was measured using a dual-luciferase reporter assay kit (GeneCopoeia, LF001).

**Co-immunoprecipitation (Co-IP) and Mass spectrometry analysis**

U87 cells (1*10^6^) were lysed in 500 µL IP buffer (Servicebio) containing 1% protease and phosphatase inhibitor, and then centrifuged at 14,000 g for 10 min to obtain protein supernatants. The protein supernatants were incubated with 5 µg Anti-PXN primary antibody (Santa Cruz, cat. no. sc-365379) at 4℃ overnight. The following day, 25 µL protein A/G Magnetic Beads (MCE) were added and incubated for 2 hours. After washing with 0.5% PBS with Tween-20 (PBST) 3 times, the beads-antibody-antigen complex was boiled with 1× loading buffer to elute the interacting protein components. Then, the samples were used for mass spectrometry analysis by Suzhou Panomik Biomedical Technology Co., Ltd.

**mRNA sequencing analysis**

mRNA sequencing analysis was conducted on U87 and U373 cells transfected with vector-shRNA and Y-box binding protein 1 (YB-1)-shRNA. Total RNA was extracted using TRIzol reagent (Beyotime) and sequenced by Shanghai Majorbio Bio-pharm Technology Co., Ltd. The data were analyzed on the online platform of Majorbio Cloud Platform^[7, 8]^. Differentially expressed genes (DEGs) were identified using DEGseq analysis, with thresholds set at |log2 ^fold change^| ≥ 1 and adjusted p-value < 0.001. GSEA software (v4.3.3) and the R package singleseqgset (v0.1.2.9000) were used for GSEA analysis. Pathways with normalized enrichment score (NES) > 1, p-value < 0.05 and q-value < 0.25 were considered significantly enriched. KEGG analysis was automatically annotated on the online platform of Majorbio Cloud Platform, selecting pathways with a p-value < 0.05 for further analysis.

**Immunofluorescence**

GBM cells were seeded onto sterile glass coverslips in 24-well plates and cultured until ~70-80% confluency. Cells were then fixed with 4% paraformaldehyde in PBS for 15 min at room temperature, followed by three washes with PBS. After permeabilization with 0.2% Triton X-100 in PBS for 10 min, cells were blocked with 5% bovine serum albumin (BSA) in PBS for 1 h at room temperature. Coverslips were incubated overnight at 4°C with primary antibodies diluted in blocking solution. After washing three times with PBS, cells were incubated with appropriate fluorophore-conjugated secondary antibodies for 1 h at room temperature in the dark. Nuclei were counterstained with DAPI (1 μg/mL) for 5 min. Finally, coverslips were mounted onto glass slides using antifade mounting medium and imaged using a confocal laser scanning microscope.**References**

1. Goldman, M.J., Craft, B., Hastie, M., Repečka, K., McDade, F., Kamath, A., Banerjee, A., Luo, Y., Rogers, D., Brooks, A.N., et al. (2020). Visualizing and interpreting cancer genomics data via the Xena platform. Nat Biotechnol *38*, 675–678.

2. Weinstein, J.N., Collisson, E.A., Mills, G.B., Shaw, K.R., Ozenberger, B.A., Ellrott, K., Shmulevich, I., Sander, C., and Stuart, J.M. (2013). The Cancer Genome Atlas Pan-Cancer analysis project. Nat Genet *45*, 1113–1120.

3. (2008). Comprehensive genomic characterization defines human glioblastoma genes and core pathways. Nature *455*, 1061–1068.

4. Zhao, Z., Meng, F., Wang, W., Wang, Z., Zhang, C., and Jiang, T. (2017). Comprehensive RNA-seq transcriptomic profiling in the malignant progression of gliomas. Scientific data *4*, 170024.

5. Zhao, Z., Zhang, K.N., Wang, Q., Li, G., Zeng, F., Zhang, Y., Wu, F., Chai, R., Wang, Z., Zhang, C., et al. (2021). Chinese Glioma Genome Atlas (CGGA): A Comprehensive Resource with Functional Genomic Data from Chinese Glioma Patients. Genomics Proteomics Bioinformatics *19*, 1–12.

6. Li, X., Wang, Y., Wu, W., Xiang, J., Wang, M., and Yu, H. (2022). A novel DNA damage and repair-related gene signature to improve predictive capacity of overall survival for patients with gliomas. Journal of cellular and molecular medicine *26*, 3736–3750.

7. Ren, Y., Yu, G., Shi, C., Liu, L., Guo, Q., Han, C., Zhang, D., Zhang, L., Liu, B., Gao, H., et al. (2022). Majorbio Cloud: A one-stop, comprehensive bioinformatic platform for multiomics analyses. iMeta *1*, e12.

8. Han, C., Shi, C., Liu, L., Han, J., Yang, Q., Wang, Y., Li, X., Fu, W., Gao, H., Huang, H., et al. (2024). Majorbio Cloud 2024: Update single-cell and multiomics workflows. iMeta *3*, e217.

**Supplementary figures and figure legends**

**
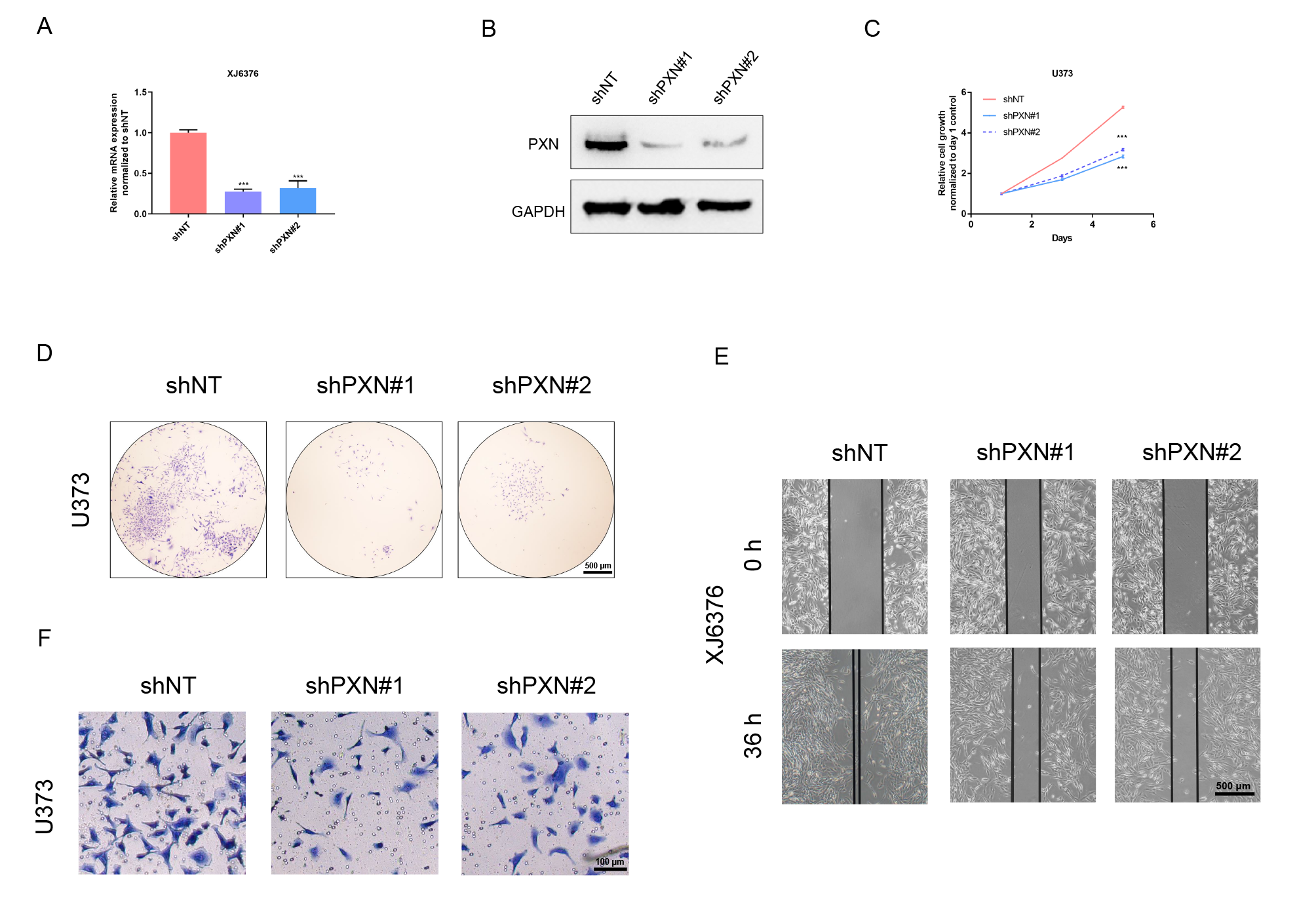
**

**Figure S1. PXN silencing hinders the proliferation, migration and tumorigenicity of GBM cells**

(**A**) qRT-PCR detecting PXN mRNA expression in XJ6376 cells transfected with NT-shRNA or PXN-shRNA. GAPDH served as a control. Data are presented as mean ± SD of three independent biological replicates. Statistical significance was determined by one-way ANOVA with Dunnett’s multiple comparisons test (***P < 0.001). (**B**) Western blotting determining PXN protein expression in XJ6376 cells transfected with NT-shRNA or PXN-shRNA. (**C**) Cell viability assay detecting the proliferation ability of U373 cells transfected with NT-shRNA or PXN-shRNA. Data are presented as mean ± SD of three independent biological replicates. Statistical significance was determined by one-way ANOVA with Dunnett’s multiple comparisons test (***P < 0.001). (**D**) Colony formation assay detecting tumorigenic potential of U373 cells transfected with NT-shRNA or PXN-shRNA. Scale bar = 500 μm. (**E**) Wound healing assay detecting the migratory ability of XJ6376 cells transfected with NT-shRNA or PXN-shRNA. Scale bar = 500 μm. (**F**) Transwell assay detecting migratory ability of U373 cells transfected with NT-shRNA or PXN-shRNA. Scale bar = 100 μm.


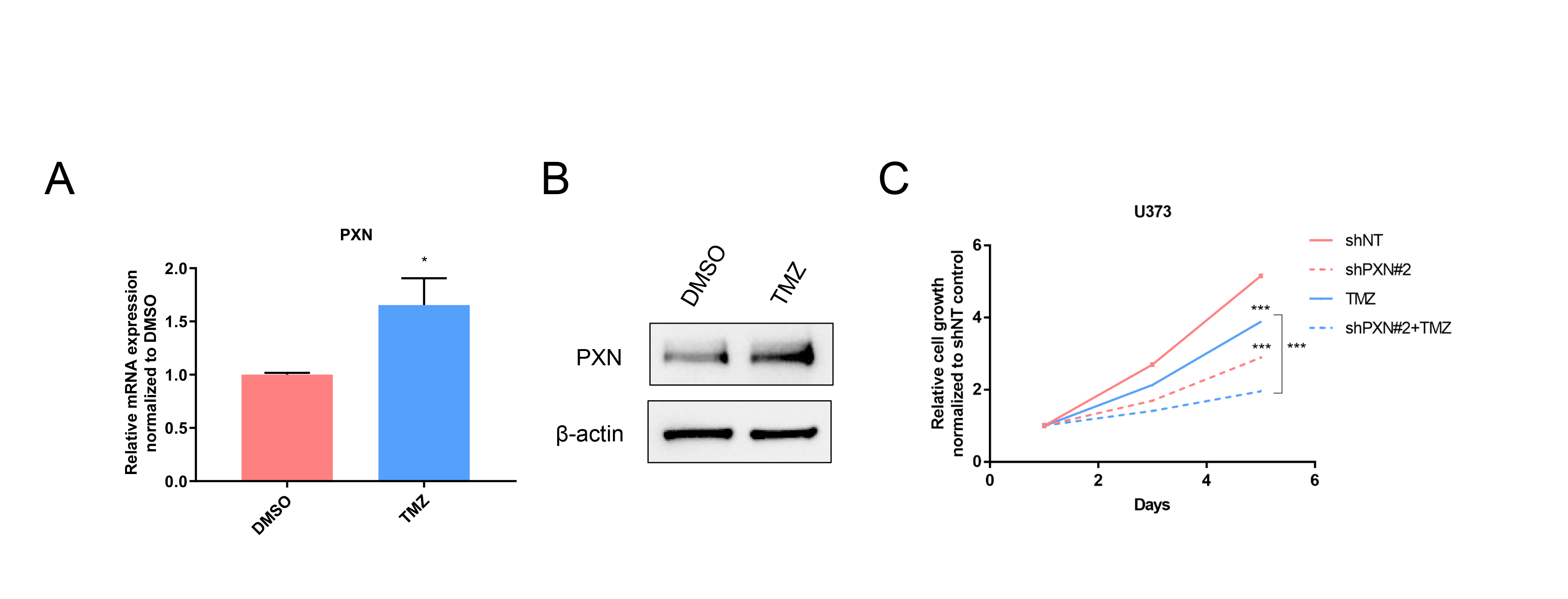


**Figure S2. Combined treatment with PXN silencing and TMZ induces increased apoptosis in GBM cells**

(**A**) qRT-PCR detecting PXN mRNA expression in XJ6376 cells pre-treated with DMSO or TMZ. Data are presented as mean ± SD of three independent biological replicates. Statistical significance was determined by a two-tailed Student’s *t*-test (***P < 0.001). (**B**) Western blotting determining PXN expression in XJ6376 cells pre-treated with DMSO or TMZ. (**C**) Cell viability assay detecting proliferation ability of U373 cells under different treatment conditions. Data are presented as mean ± SD of three independent biological replicates. Statistical significance was determined by one-way ANOVA with Tukey's post hoc test (***P < 0.001).


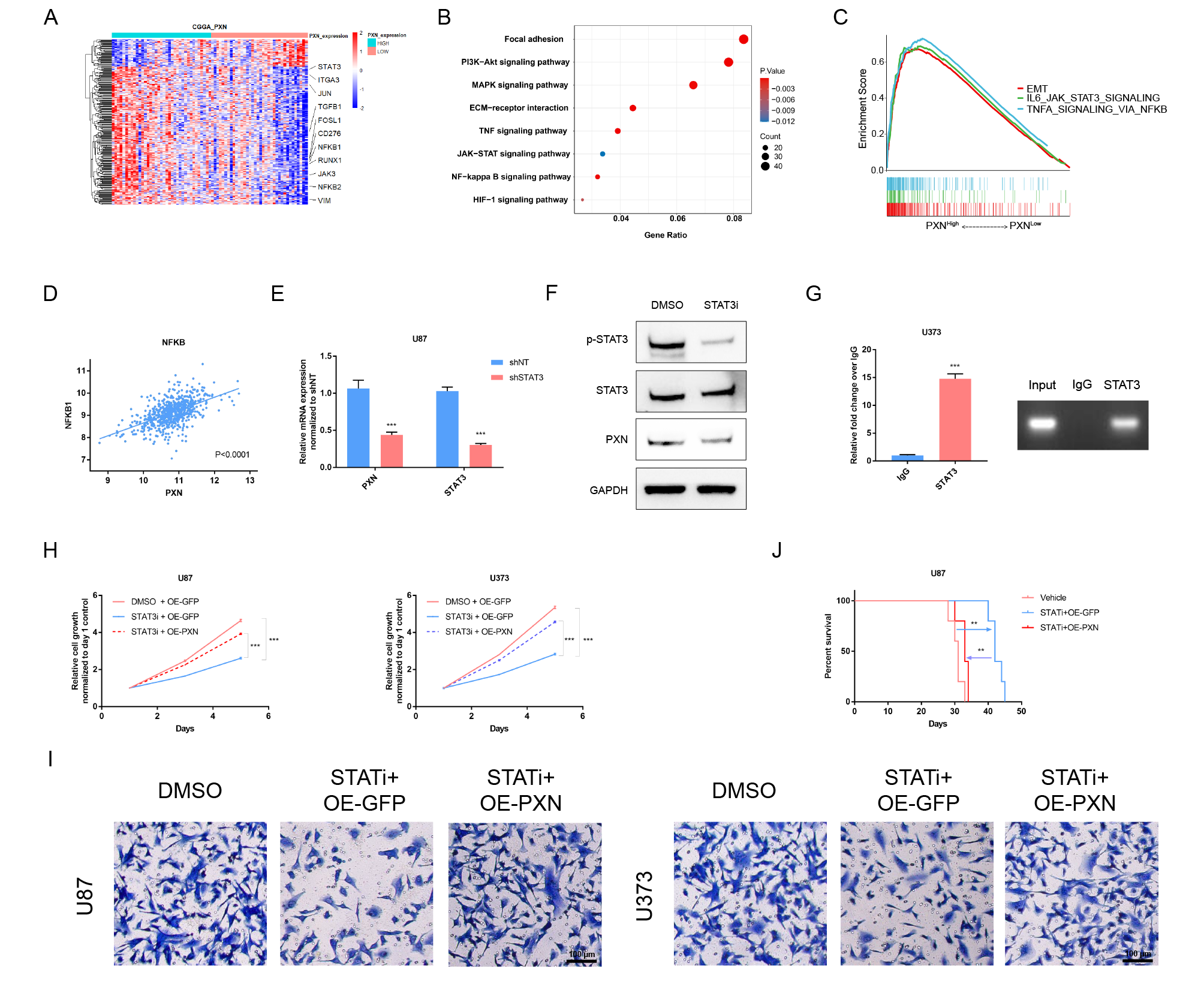


**Figure S3.** **STAT3 acts as an upstream factor of PXN to drive GBM progression**

(**A**) A heatmap showing DEGs between the PXN^High^ and PXN^Low^ groups using information from CGGA dataset. (**B**) KEGG analysis showing pathways enriched in the PXN^High^ group. (**C**) GSEA showing oncogenic pathways enriched in the PXN^High^ group. (**D**) Correlation analysis of mRNA expression relationship of PXN with NFKB1. Pearson’s correlation coefficient (r) was calculated to assess the linear relationship, with statistical significance determined by a two-tailed Student’s *t*-test. (**E**) qRT-PCR showing PXN mRNA expression in U87 cells transfected with NT-shRNA or STAT3-shRNA. Data are presented as mean ± SD of three independent biological replicates. Statistical significance was determined by a two-tailed Student’s *t*-test (***P < 0.001). (**F**) Western blotting detecting STAT3, p-STAT3 and PXN protein expression in XJ6376 cells pre-treated with DMSO or STAT3i. (**G**) ChIP-PCR detecting enrichment of STAT3 at PXN promoter in U373 cells, and representative agarose gel electrophoresis images of corresponding ChIP samples amplified using PXN promoter-specific primers. Data are presented as mean ± SD of three independent biological replicates. Statistical significance was determined by a two-tailed Student’s *t*-test (***P < 0.001). (**H**) Cell viability assay detecting proliferation ability of U87 and U373 cells subjected to the indicated treatments. Data are presented as mean ± SD of three independent biological replicates. Statistical significance was determined by one-way ANOVA with Dunnett’s multiple comparisons test (***P < 0.001). (**I**) Transwell assay detecting migratory ability of U87 and U373 cells subjected to the indicated treatments. Scale bar = 100 μm. (**J**) K-M analysis comparing overall survival of mice intracranially injected with U87 cell subjected to the indicated treatments. Data are presented as median survival time (n = 5 mice per group). Statistical significance was determined by log-rank test (**P < 0.01).

**Figure S4. PXN Nuclear Localization and Its Role in STAT3 Activation in GBM Cells**

(**A**) Western blot analysis of STAT3 and p-STAT3 in U373 cells treated with the SRC inhibitor (MCE, HY-101053) at 2 μM, 5 μM, and 10 μM for 2 h. (**B**) Nuclear-cytoplasmic fractionation followed by western blotting reveals subcellular localization of p-PXN in U87 and U373 cells. (**C**) Western blot analysis of p-PXN in U87 cells following 30-min stimulation with 20 ng/mL EGF (TargetMol, P01133). (**D**) Immunofluorescence reveals subcellular localization of p-PXN in U87 and U373 cells under control conditions or after 30-min EGF stimulation (20 ng/mL). Scale bar = 20 μm.

**
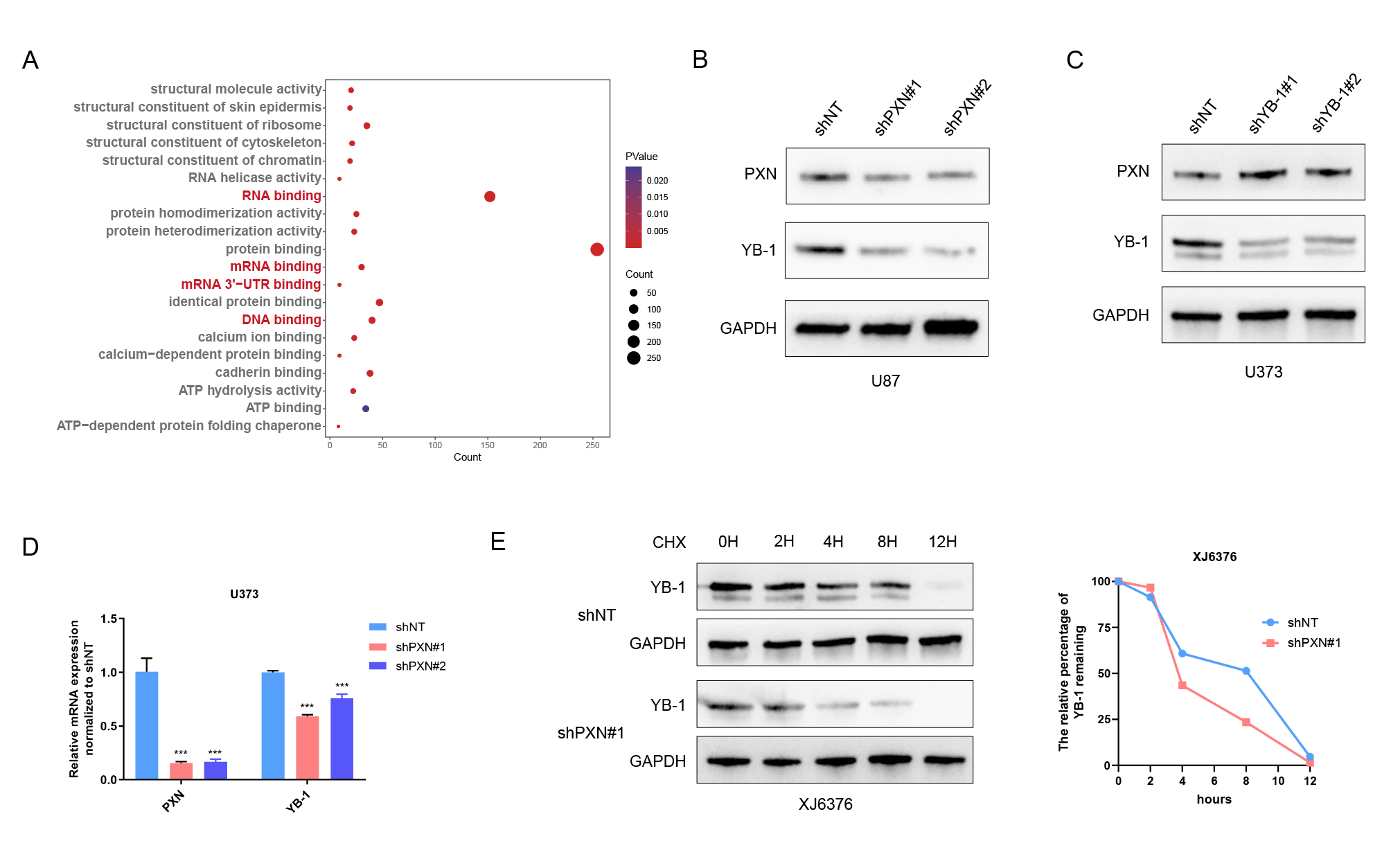
**

**Figure S5. PXN enhances YB-1 stability by inhibiting ubiquitin-mediated degradation**

(**A**) A bubble plot showing top 20 Gene Ontology (GO) results of identified proteins by mass spectrometry analysis using the DAVID website. (**B**) Western blotting determining YB-1 protein expression in U87 cells transfected with NT-shRNA or PXN-shRNA. (**C**) Western blotting determining PXN protein expression in U373 cells transfected with NT-shRNA or YB-1-shRNA. (**D**) qRT-PCR detecting YB-1 mRNA expression in U373 cells transfected with NT-shRNA and PXN-shRNA. Data are presented as mean ± SD of three independent biological replicates. Statistical significance was determined by a one-way ANOVA with Dunnett’s multiple comparisons test (***P < 0.001). (**E**) Western blotting comparing YB-1 protein degradation rate in XJ6376 cells transfected with NT-shRNA and PXN-shRNA.
