# Supplementary material for: Deciphering the STAT3-PXN positive feedback loop in GBM, IDH-wildtype: transcriptional regulation and inhibition of YB-1 ubiquitination
Source: Cell Death Discov. 2026 Mar 23;12:168. doi: 10.1038/s41420-026-03035-9 (PMC13039979; doi:10.1038/s41420-026-03035-9)

Figure 1E

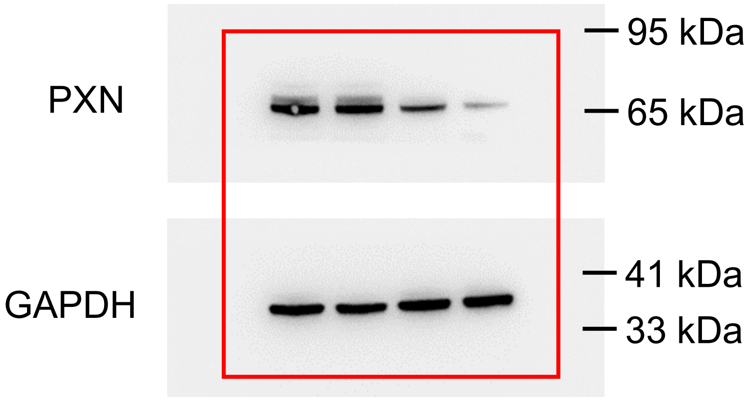

Figure 1G

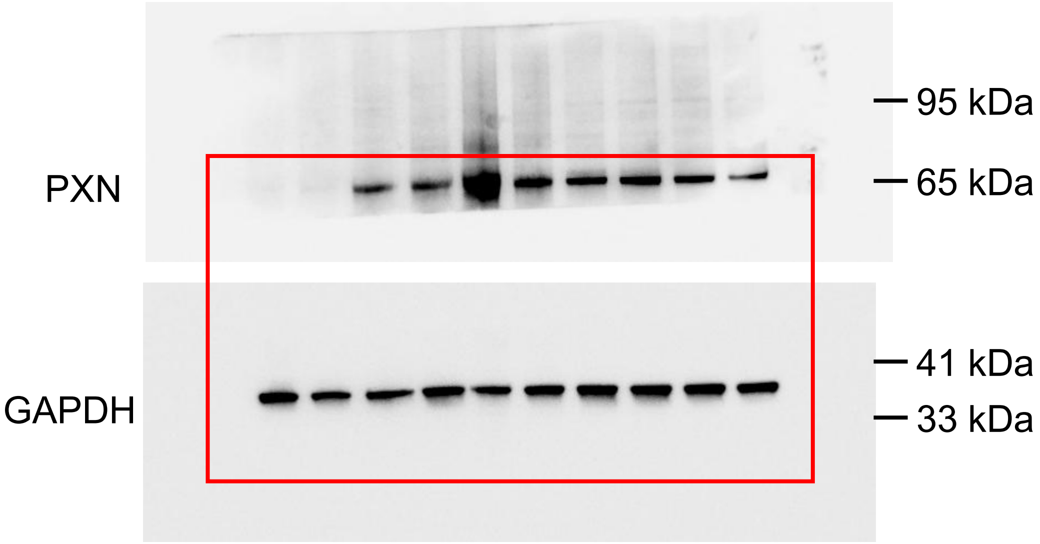

Figure 2B

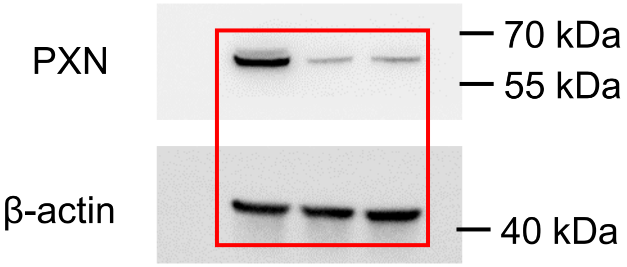

Figure S1B

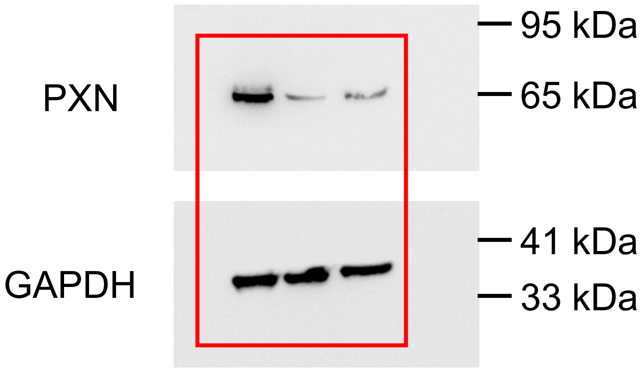

Figure 3B

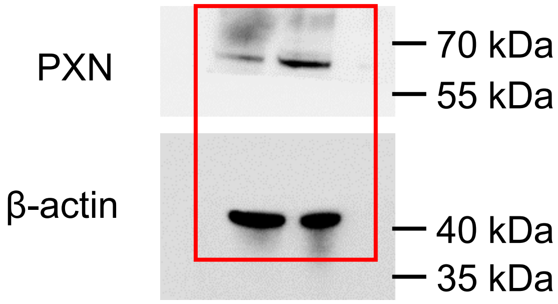

Figure 4B

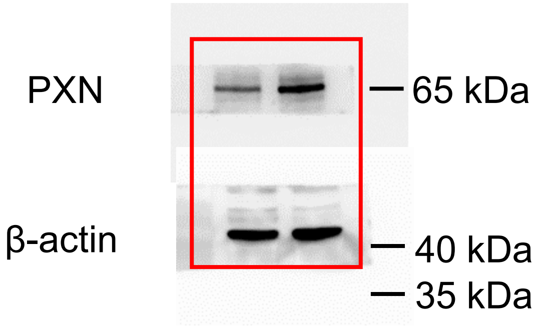

Figure S2B

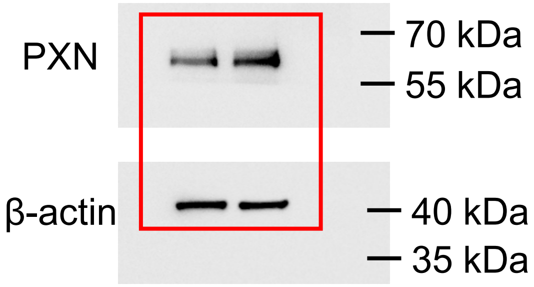

Figure 4E

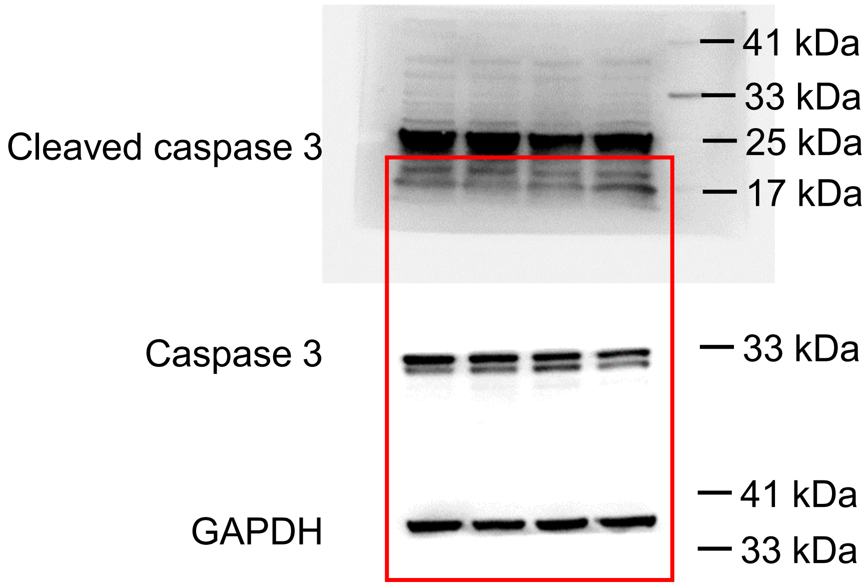

Figure 5G

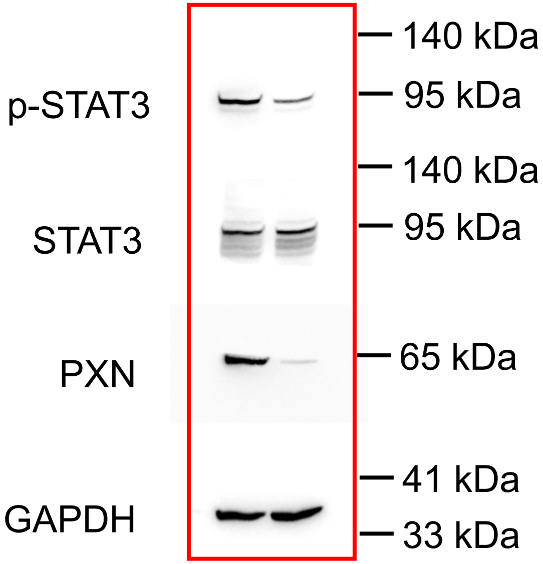

Figure S3F

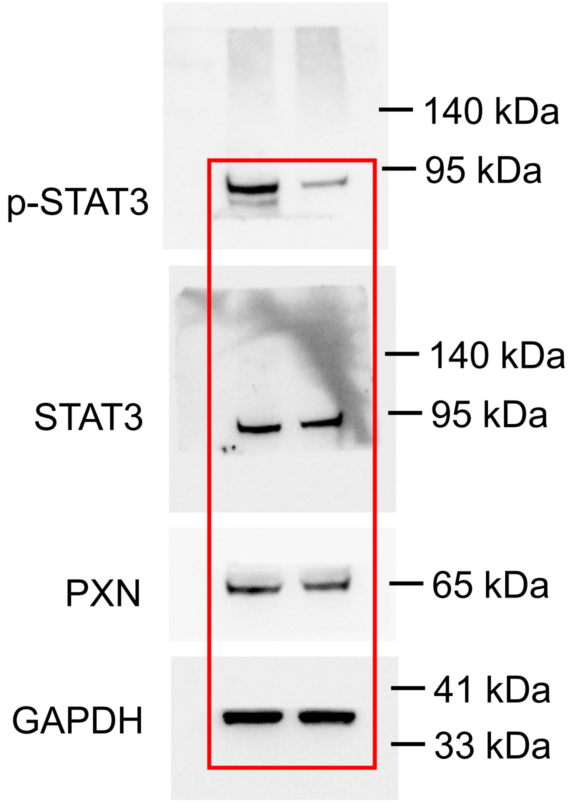

Figure 6A

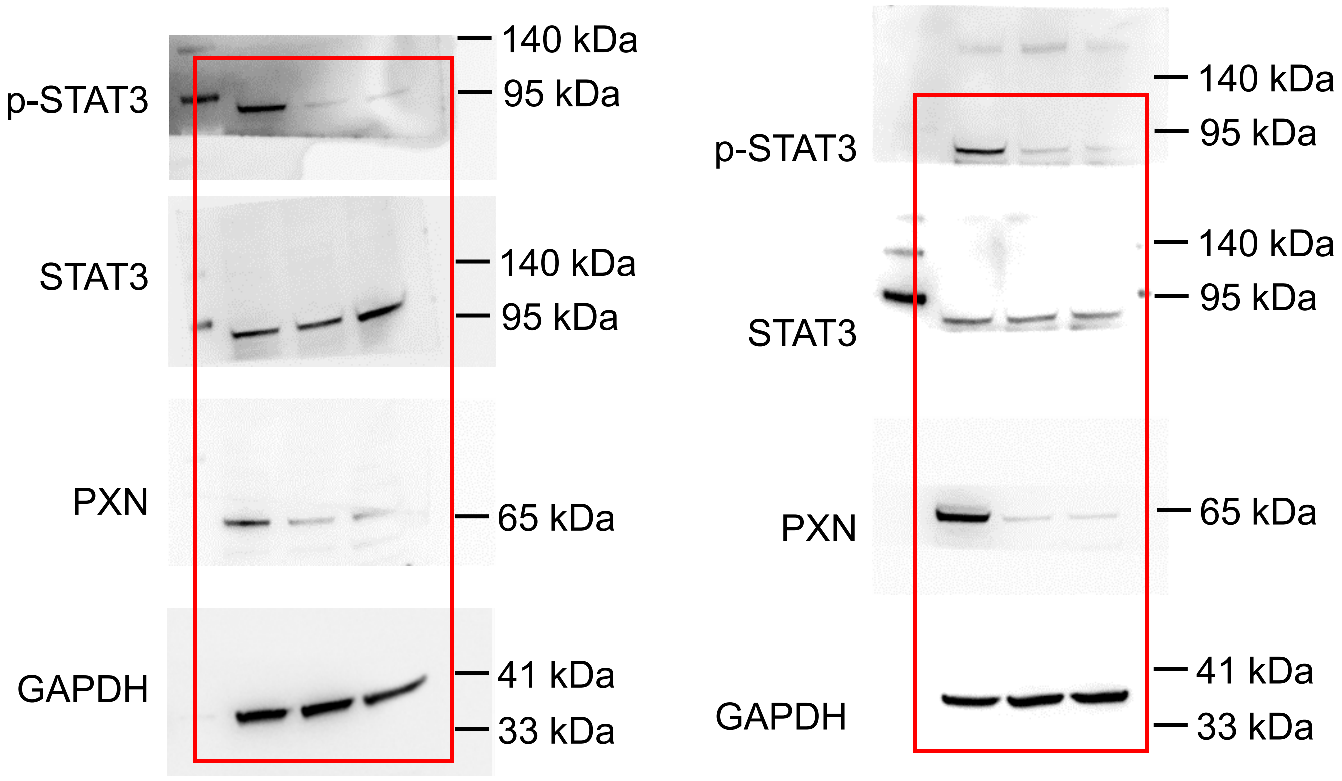

Figure 6D

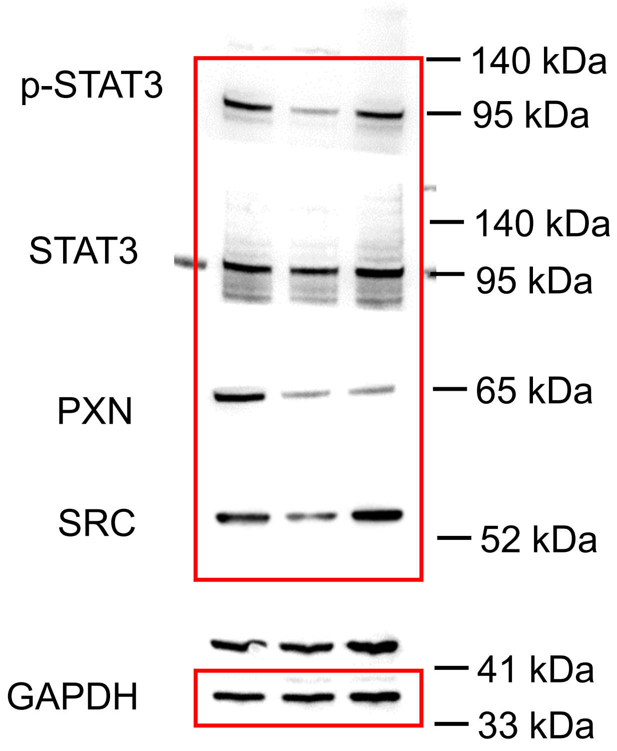

Figure S4A

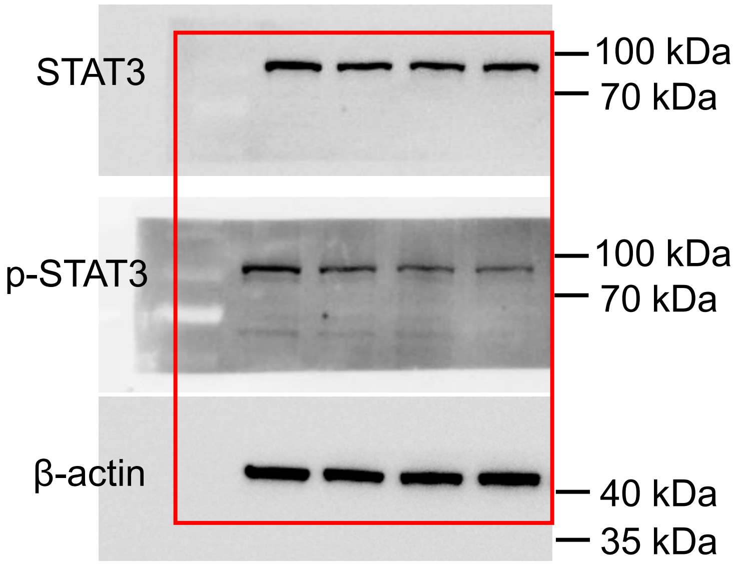

Figure S4B

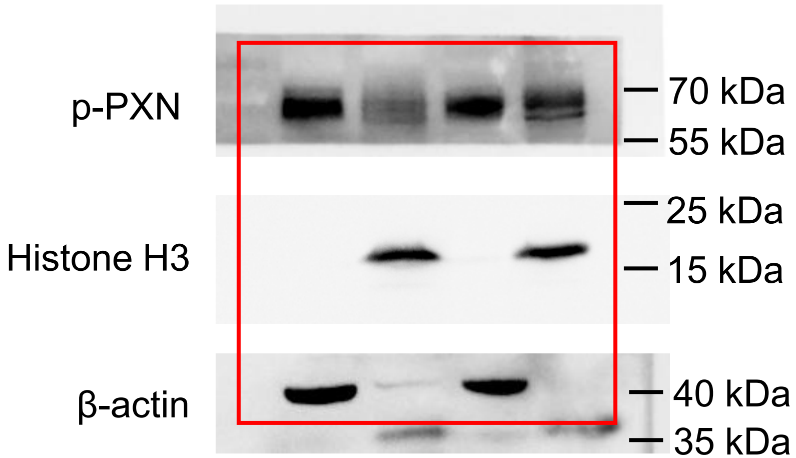

Figure S4C

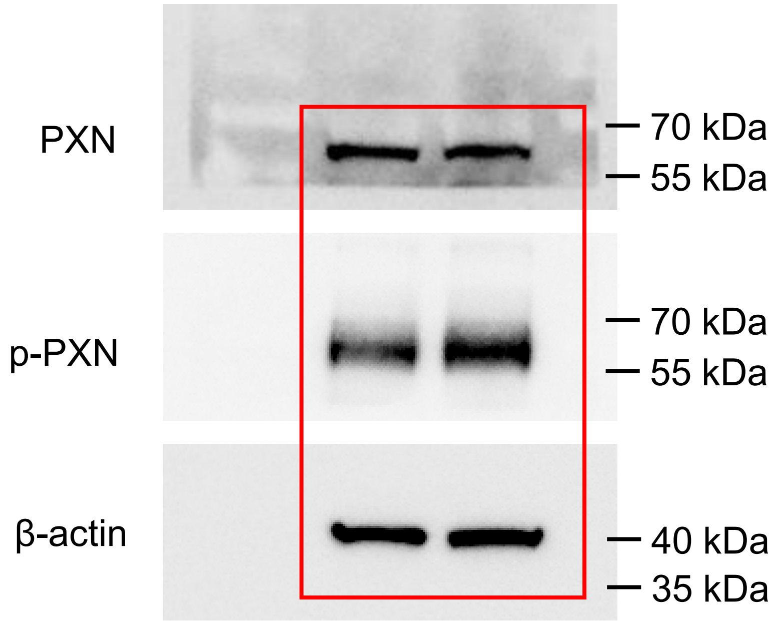

Figure 7A

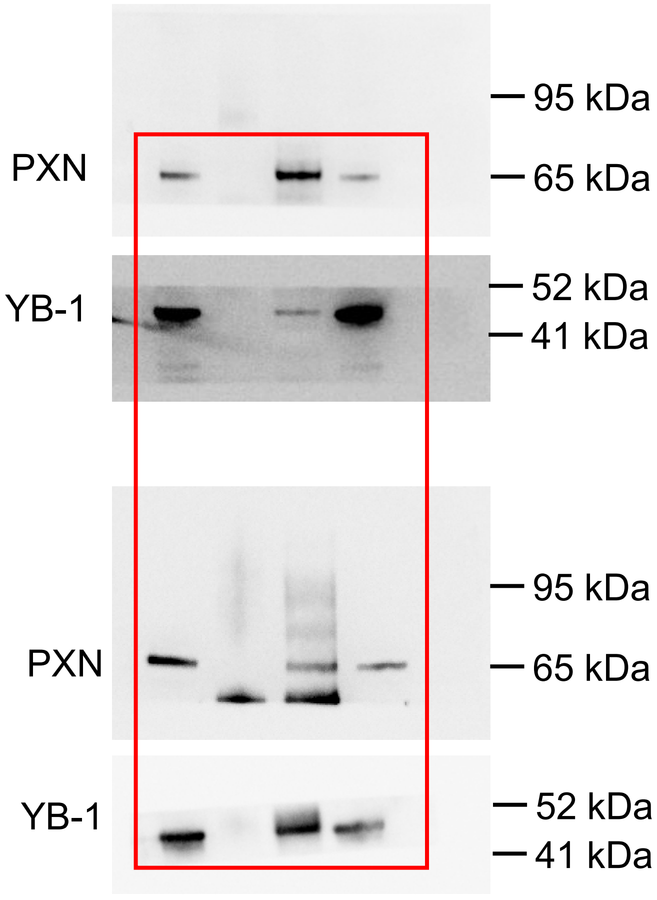

Figure 7B

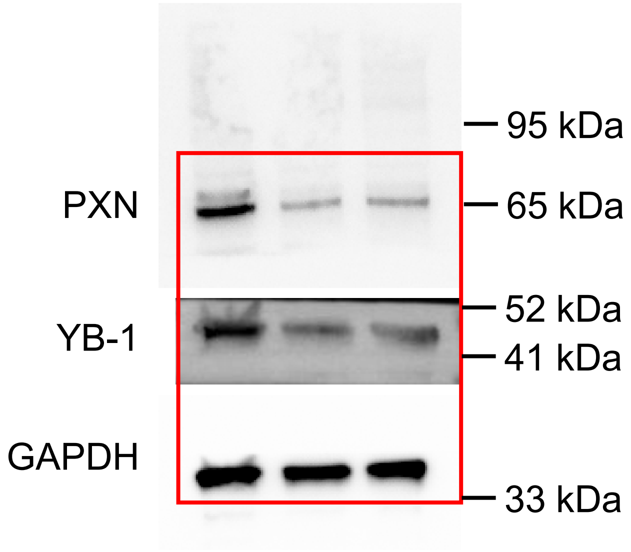

Figure 7C

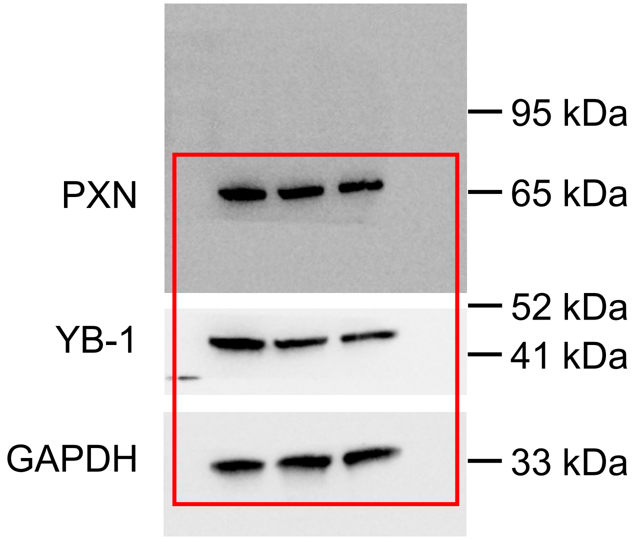

Figure S5B

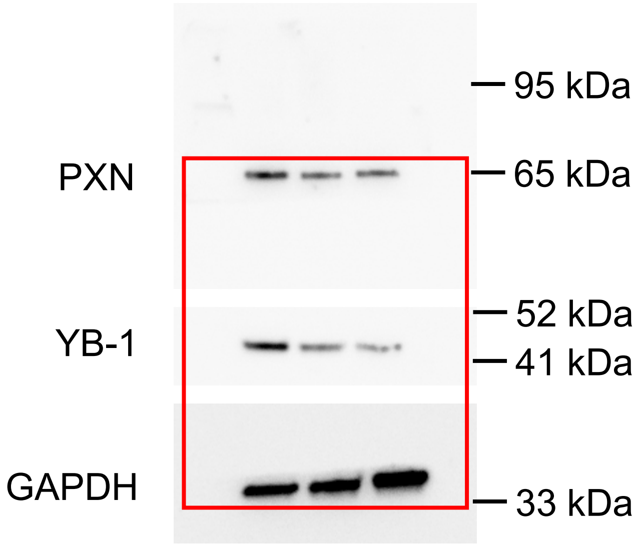

Figure S5C

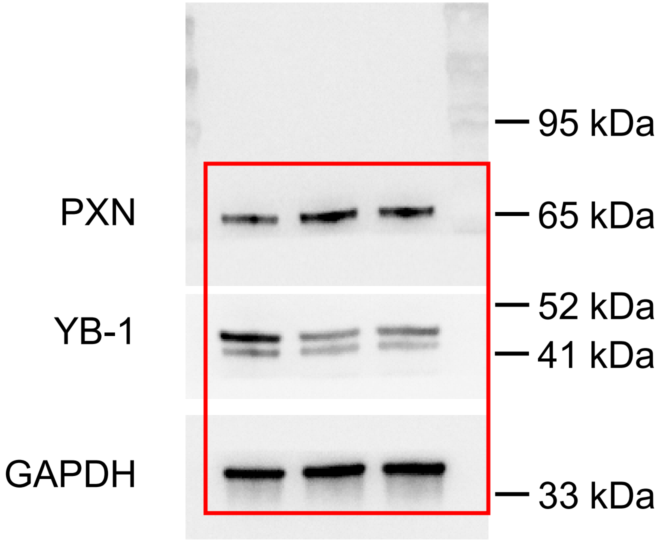

Figure 7E

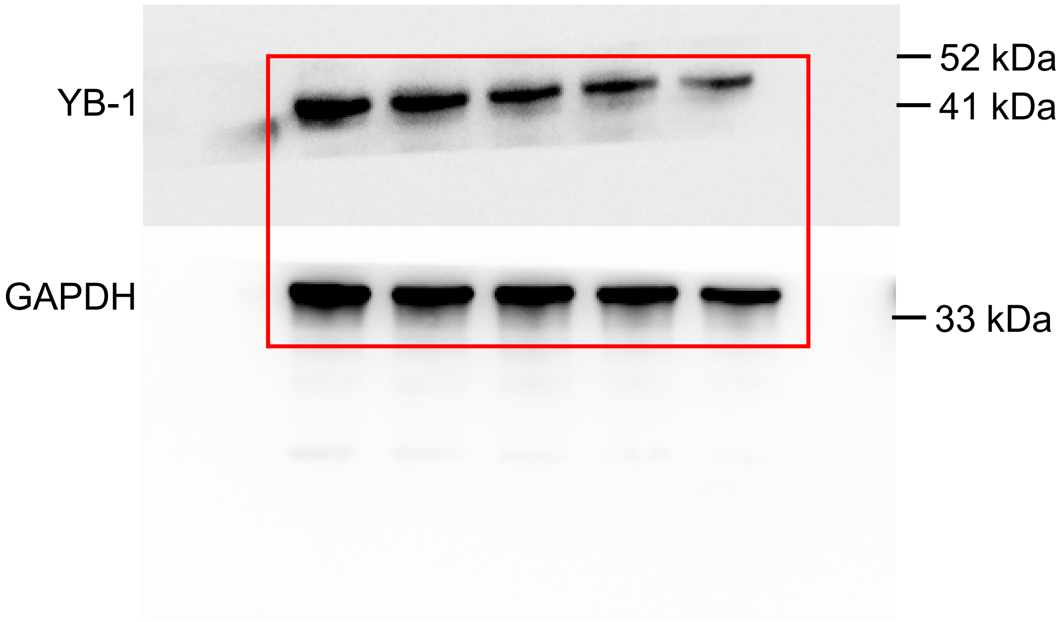

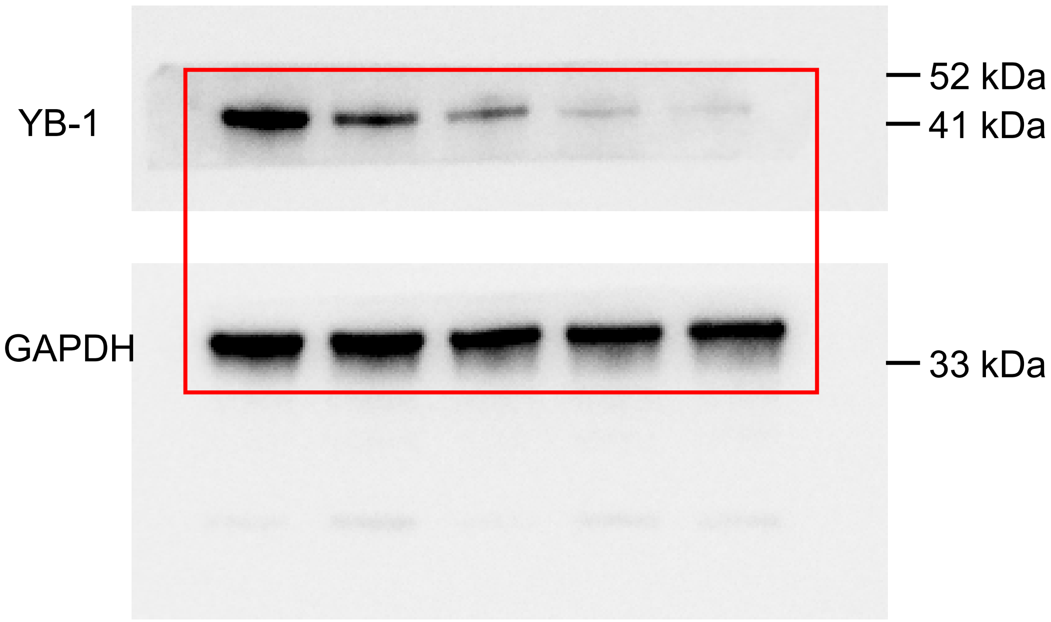

Figure S5E

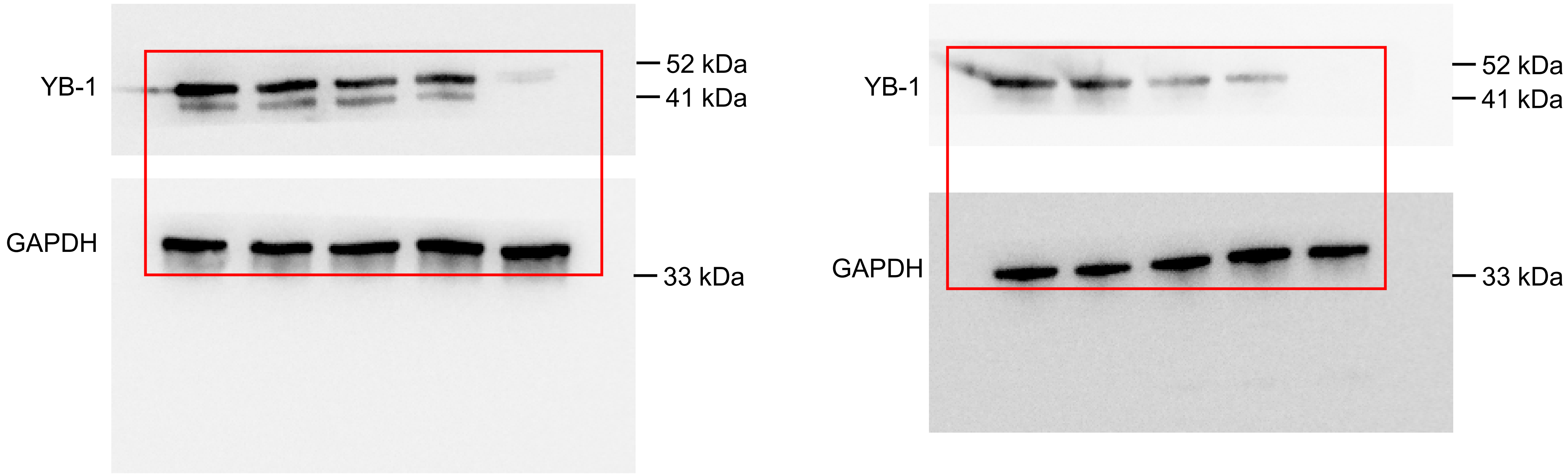

Figure 7F

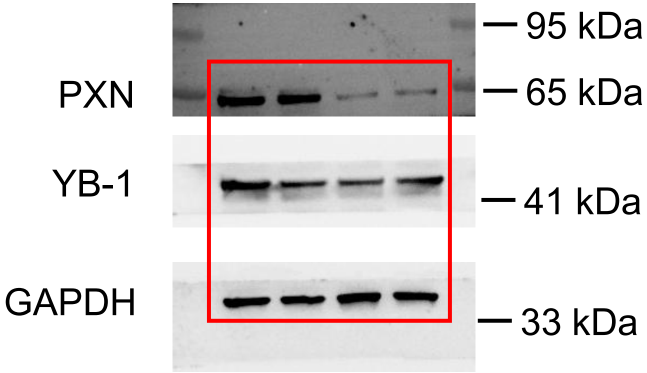

Figure 7G

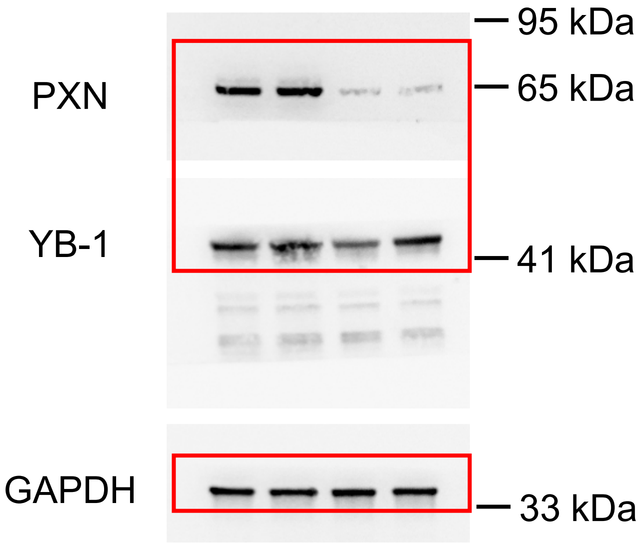

Figure 7H

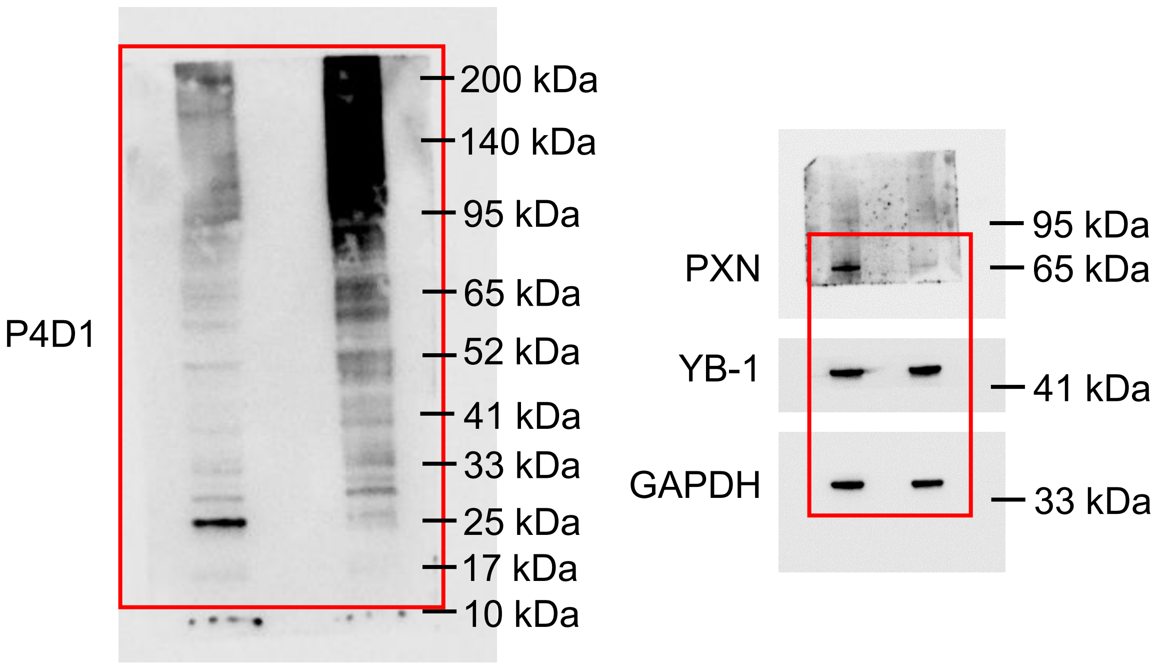

Figure 7I

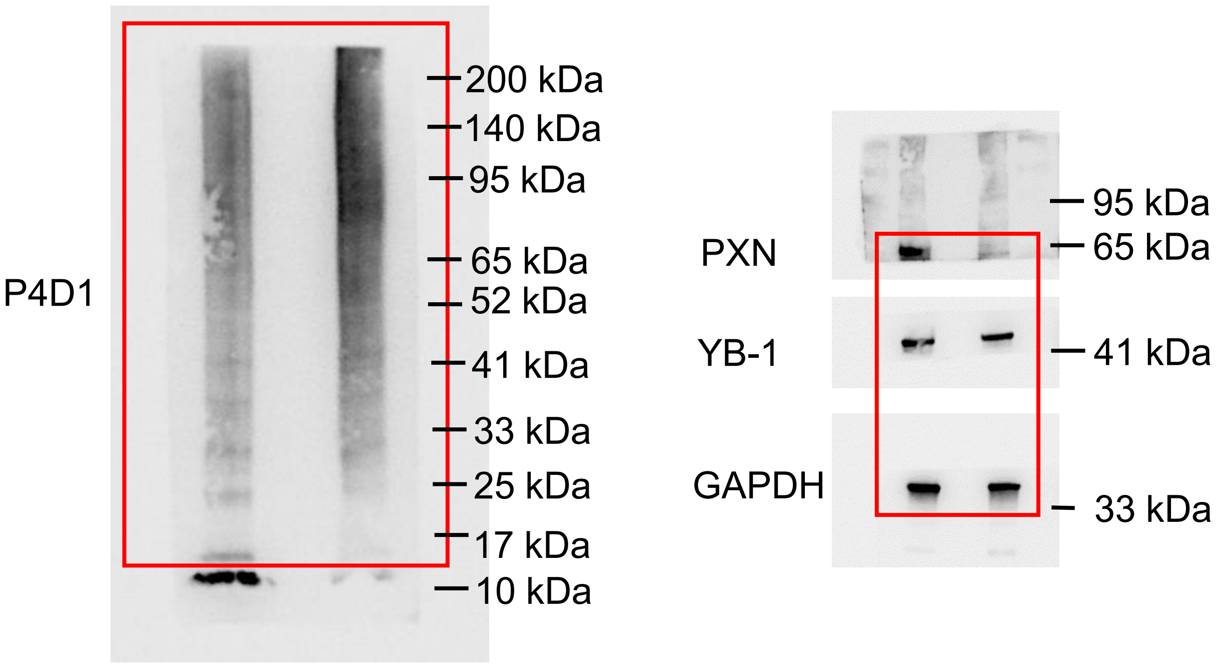

Figure 7J

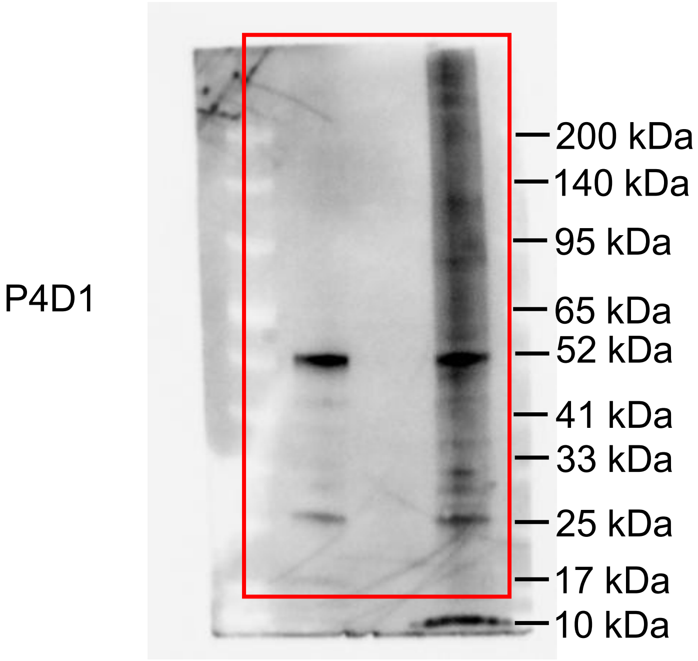

Figure 7K

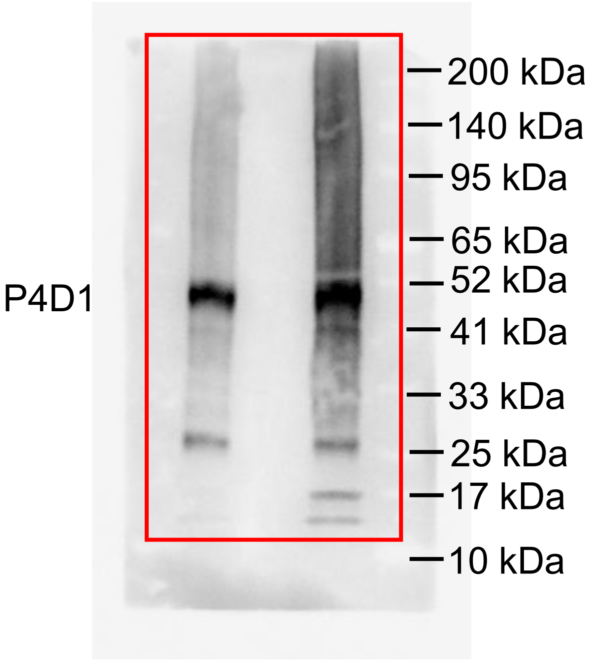

Supplement: Supplementary file 3 — Original western blots [file 41420_2026_3035_MOESM3_ESM.pdf]
